# Surface Passivation of HgTe Nanocrystals Enabling E_G_/2 Open‐Circuit Voltage and Their Coupling to Dielectric Cavity for Narrow Detection

**DOI:** 10.1002/adma.73019

**Published:** 2026-04-10

**Authors:** Albin Colle, Clement Gureghian, Dario Mastrippolito, Mariarosa Cavallo, Jiho Roh, Marco Paye, Tommaso Gemo, Diogo Almeida, Adrien Khalili, Yoann Prado, Xavier Lafosse, Sandrine Ithurria, Mathieu G. Silly, Pavel Dudin, James K. Utterback, José Avila, Debora Pierucci, Emmanuel Lhuillier

**Affiliations:** ^1^ Institut des NanoSciences de Paris CNRS Sorbonne Université Paris France; ^2^ LYNRED Actipole – CS 10021 Veurey‐Voroize France; ^3^ Centre de Nanosciences et de Nanotechnologies CNRS Université Paris‐Saclay Palaiseau France; ^4^ Laboratoire de Physique et d'Etude des Matériaux CNRS ESPCI, PSL Research University Sorbonne Université Paris France; ^5^ Synchrotron SOLEIL L'Orme des Merisiers Saint‐Aubin France

**Keywords:** core–shell structure, dielectric cavity, infrared detection, nanocrystals, photoemission microscopy, surface passivation

## Abstract

Colloidal HgTe nanocrystals (NCs) offer a versatile, solution‐processable platform for infrared optoelectronics, yet their integration into high‐performance diodes has long been hindered by surface‐trap‐limited open‐circuit voltage (*V*_OC_), high dark currents, and insufficient thermal robustness. Here, we demonstrate that ultrathin CdS shells grown around HgTe cores, combined with an optimized cation‐exchange protocol, enable unprecedented passivation of trap states while reducing species interdiffusion and simultaneously improving interfacial band alignment. Implemented in a diode architecture employing SnO_2_ electron‐transport layers and Ag‐doped CdTe hole‐selective contacts, these HgTe/CdS NCs yield a two orders of magnitude reduction in dark current and a *V*_OC_ of 420 mV; exceeding half the optical bandgap for the first time in HgTe‐based NC photodiodes. Operated at room temperature, the devices exhibit detectivities up to 1.5 × 10^1^ ^1^ Jones and fast response times below 200 ns. Leveraging the reduced dark current and improved film homogeneity, we further integrate the photodiodes into a dielectric Bragg cavity to achieve ultranarrow detection linewidths down to 90 cm^−1^ at 1.55 µm. This diode design benefits from a strong field enhancement, while the device absorption limits the linewidth. Our results establish surface‐passivated HgTe NCs as a viable route toward compact, narrowband, and thermally stable infrared photodetectors.

## Graphical abstract

This article explores how the integration of a thin shell, combined with optimized surface chemistry, enables the design of photodiodes based on short‐wave infrared HgTe nanocrystals. These photodiodes achieve an open‐circuit voltage greater than half the optical bandgap, which drastically reduces dark current. The article also explores the integration of the diode into a dielectric cavity to narrow the spectral response.

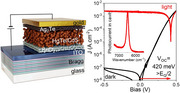

## 1 Introduction

Colloidal HgTe [1, 2] nanocrystals (NCs) [3], have emerged as one of the most advanced solution‐processed materials for infrared (IR) optoelectronics. Owing to the semi‐metallic character of bulk HgTe, their optical response can be tuned over more than two orders of magnitude in photon energy, enabling continuous spectral coverage from the visible to the terahertz regime [4, 5]. In addition, HgTe NCs exhibit markedly higher resistance to oxidation [6] compared to other colloidal IR materials such as PbS, PbSe, and III–V NCs, providing an important material advantage for device stability.

Significant progress has recently been made along two complementary directions. On the photonics side, substantial efforts have focused on coupling HgTe NC absorbers to engineered optical structures [7, 8, 9] to overcome the mismatch between the short carrier diffusion length, limited to only a few nanocrystals, and the much larger optical absorption depth, which can reach several micrometers. By confining and enhancing the local optical field within thin NC layers where charge transport remains efficient, these photonic architectures not only increase absorption [10, 11], but also provide additional control over spectral selectivity [12].

From a device‐engineering perspective, new architectures [13] have been introduced to increase the built‐in electric field and improve charge separation. In the short‐wave infrared (SWIR) range, electron‐transport layers based on Bi_2_Se_3_ [14], SnO_2_ [15], or CdSe [16], have significantly boosted device performance. Concurrently, strategies based on ligand exchange [17], controlled doping [18] or alloying [19] have enabled the formation of homojunctions [8, 10, 20, 21] in HgTe NC films.

The rapid improvement in performance has facilitated a transition from single‐pixel devices to full‐format imagers [13, 17, 22, 23]. However, this scale‐up has introduced new challenges: the ≈10^5^‐fold increase in pixel count dramatically raises Joule heating within the focal plane. Although HgTe NCs are relatively resistant to oxidative degradation, their thermal stability remains limited [6, 24], particularly for SWIR NCs synthesized only slightly above room temperature (≈60°C). In passive systems without dedicated thermal management, heating from the readout integrated circuit (ROIC) can induce nanocrystal sintering, leading to substantial redshifts of the absorption edge and, more critically, a sharp rise in dark current due to bandgap narrowing. This thermal vulnerability represents a major bottleneck for industrial translation.

**FIGURE 1:**
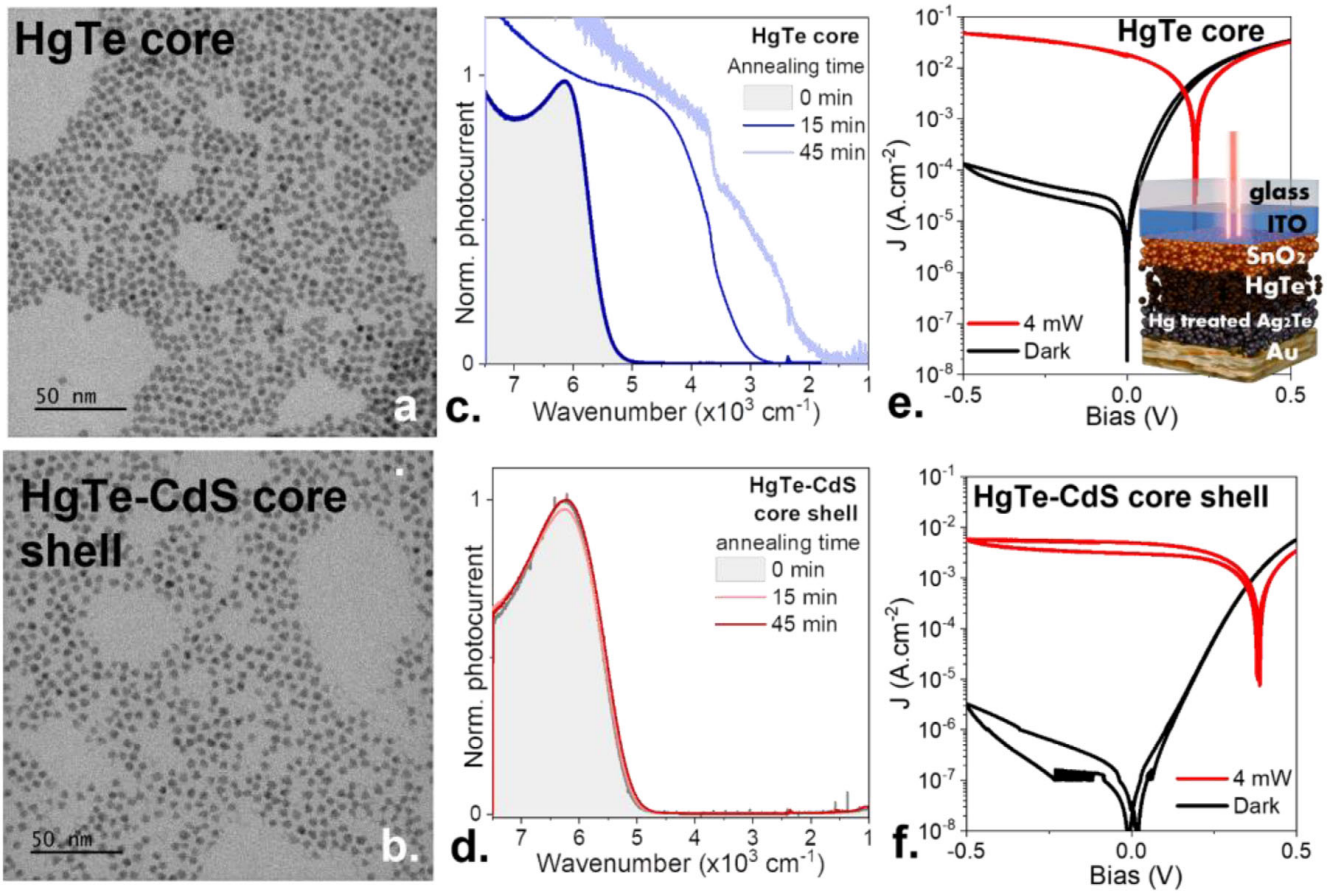
HgTe‐CdS NCs and their benefits for photodiode. (a) (resp. (b)) TEM image of HgTe core (resp. HgTe/CdS). (c) (resp. (d)) Photocurrent spectra after various times of annealing at 100°C for core only (resp for HgTe/CdS) NCs. (e) (resp. (f)) Current density as a function of the applied bias in the dark and under illumination for core only (resp for HgTe/CdS) NCs. The inset of part (e) is a schematic of the diode stack. Note that the noise on the I‐V curve at 10^−7^ A cm^−2^ is due to the change of bandwidth of the instrument as the current gets below the nA range.

Zhang et al. recently proposed an elegant solution based on the growth of a thin CdS shell [25] (Figure 1b). A shell thickness of roughly one monolayer is sufficient to dramatically enhance thermal stability (Figure 1d), while remaining thin enough to avoid the formation of a large tunnel barrier that would impede charge transport. This core/shell architecture enables improved imaging stability and reduced dark current, thereby permitting longer integration times and improved image contrast [25]. However, to date, these benefits have only been demonstrated in photoconductive configurations, while photovoltaic operation of this platform has not yet been reported.

A second, broader challenge in HgTe NC photodiodes relates to dark current and the associated open‐circuit voltage (*V*_OC_). Relative to photoconductors, diodes naturally benefit from their built‐in field and can operate at/or near zero bias, reducing dark current. Yet in SWIR HgTe photodiodes, reported *V*_OC_ values consistently saturate at ≈200–250 mV (see Table 1; Figure S1), corresponding to only *E*_G_/4–*E*_G_/3 for this spectral range, where *E*_G_ is the band edge energy. This is clearly below the state‐of‐the‐art PbS NCs, even though this comparison has its limit due to inhomogeneity of bias, irradiance and cut‐off bandgap (1.7–2.2 µm range). For example, PbS NCs with a ≈950 nm bandgap routinely reach *V*_OC_ ≈ *E*_G_/2 [26, 27], and perovskite NCs, known for their defect tolerance, often exhibit *V*_OC_ values exceeding 60% of the bandgap [28, 29]. Because surface traps and incomplete passivation are well‐established sources of *V*_OC_ loss in NC solids [30], improving surface chemistry appears essential for further progress in HgTe‐based photodiodes.

**TABLE 1:** Figures of merit relative to light detection for photodiodes based on HgTe NCs and operating in the SWIR.

Stack	Dark current (A cm^−2^)	Responsivity (A/W)	*V*_OC_ (mV)	Detectivity (Jones)	λ_cut‐off_ (µm)	Time response (ns)	Refs.
FTO/CdSe/HgTe/Ag_2_Te/Au	7 × 10^−3^ @ 0.4 V	0.8	250	9 × 10^11^ @200K, 9 × 10^10^ @300 K	2.0	200–700	[16]
FTO/SnO_2_/HgTe/Ag_2_Te/Au	2 × 10^−4^ @ 0.4 V	0.3	230	5 × 10^10^ @ 300 K	2.0	300	[15]
ITO/ZnO/HgTe/ZnTe/Au	2 × 10^−6^ @ 0.4 V	0.72	190	3.6 × 10^11^ @ 300 K	1.7	580	[13]
ITO/Bi_2_S_3_/Se:HgTe/Au:HgTe/Au	7 × 10^−7^ @ 0.4 V	0.76	200	1.6 × 10^12^ @ 300 K	1.8	2200	[18]
ITO/Bi_2_S_3_/Se:HgTe/Ag_2_Te/Au	3 × 10^−6^ @ 0.4 V	0.69	150	5.2 × 10^11^ @ 300 K	2.2	2600	[19]
ITO/SAM/Ag_2_Te/Se:HgTe/Bi_2_S_3_/ITO	4.6 × 10^−7^ @ 0.4 V	0.76	200	2.1 × 10^12^ @ 300 K	1.9	6400	[40]
ITO/Bi_2_S_3_/HgTe/Ag_2_Te/Au	2.3 × 10^−6^ @ 0.4 V	0.38	220	3.9 × 10^11^ @ 300 K	1.7	25 400	[41]
ITO/Bi_2_S_3_/HgTe/Ag_2_Te/Au	3 × 10^−7^ @ 0.4 V	0.5	250	8.1 × 10^11^ @ 300 K	1.7	16 000	[42]
ITO/HgTe/Ag_2_Te/Au	4 × 10^−6^ @ 0.3 V	0.24	50	2.7 × 10^10^ @ 300 K	2.8	390	[43]
ITO/Blend Ag_2_Te‐HgTe/HgTe/Bi_2_S_3_/ITO	3 × 10^−6^ @ 0.4 V	0.62	160	3.4 × 10^11^ @ 300 K	1.9	19 000	[44]
ITO/HgTe/Ag_2_Te/Au	4 × 10^−6^ @ 0.4 V	1	50	1 × 10^11^ @ 300 K	2.2	1400	[45]
ITO/Au‐nanorods/ZnO/HgTe/MoO_3_/Au	4 × 10^−6^ @ 0.1 V	/	210	1.4 × 10^10^ @ 300 K	1.4	/	[46]
Graphene/Bi_2_S_3_/HgTe/Ag_2_Te/Au	/	0.3	/	5 × 10^9^ @ 300 K	2.4	13	[47]
ITO/PbS‐MP/PbS‐MAPbI_3_/ZnO/Au	8 × 10^−6^ @ 0.5 V	0.78	350	2.5 × 10^12^ @ 300 K	1.5	6000	[48]
ITO/ZnO/Halide‐PbS/EDT‐PbS/Au	3 × 10^−6^ @ 0.5 V	1	340	8 × 10^11^ @ 300 K	1.7	10	[49]
ITO/SnO_2_/Ag_2_Te‐AgNO_3_/Ag_2_Te‐AgNO_3_ + EDT/Au	4.5 × 10^−7^ @ 0.5 V	0.25	/	1.1 × 10^11^ @ 300 K	1.6	25	[50]
ITO/NiO/InAs/Nb:TiO_2_/Al	5 × 10^−7^ @ 0.5 V	0.03	270	1.1 × 10^10^ @ 300 K	1.4	1600	[51]
ITO/ZnO/PbS‐CdS/PbS‐EDT/MoO_x_/Ag	1.9 × 10^−7^ @ 0.5 V	0.47	340	5.1 × 10^12^ @ 300 K	1.4	/	[52]
ITO/SnO_2_/HgTe‐CdS/Ag_2_Te/Au	10^−7^ @ 0.5 V		420	1.5 × 10^11^ @ 300 K	1.8	200	This work

In this work, we demonstrate the integration of HgTe/CdS core/shell NCs into photodiodes as a strategy to simultaneously address the thermal‐stability limitation and the low *V*_OC_ bottleneck. Through optimized surface passivation, we achieve a twofold increase in *V*_OC_ compared to core‐only HgTe NCs with matched bandgaps. Finally, we explore the potential of these low‐dark‐current diodes for narrowband detection near telecom wavelengths, relevant for spectroscopy and low‐background light detection and ranging (LiDAR). By combining the optimized NC film with a dielectric‐mirror cavity, we achieve ultranarrow detection bandwidths as small as 90 cm^−^^1^ (≈12 meV at 1.55 µm), approaching the intrinsic absorption linewidth of the material.

## 2 Discussion and Results

We start by growing HgTe NC cores using the procedure from Keuleyan et al. [31]. The reaction conditions are chosen to generate an excitonic feature at 6000 cm^−1^ (≈0.75 eV, see Figure S2) as a strategy extensively used for HgTe NC‐based devices. We also grow core–shell material following the procedure of Zhang et al. [25]. through decomposition of a monomolecular precursor (Cadmium bis(phenyldithiocarbamate)) on top of sphere‐shaped HgTe NCs. The shell growth (confirmed by XPS in Figure S3) induces a small redshift compared to the core (500 cm^−1^) that results from an increased size. We account for this shift by growing a slightly smaller core (Figure 1a), so that the final core–shell material presents a similar bandgap as the reference core (Figure S2). We have checked that the core–shell structure presents the expected thermal stability upon exposure to mild annealing (100°C) and observe a strong red shift only in the case of the core‐only system, see Figure 1c, with none iobserved for CdS shelled NCs, see Figure 1d.

We then integrate the material into a diode stack made of ITO/SnO_2_/HgTe/Ag_2_Te:Hg/Au as proposed by Greboval et al. [15], see the inset of Figure 1e. In this diode stack, the ITO layer is chosen to be thin (50 nm) to maintain a reasonable transparency in the SWIR range. The SnO_2_ plays the role of the electron transport layer. The absorbing HgTe layer is obtained from an ink in which the native dodecanethiols are replaced by shorter thiols (mercaptoethanol: MPOH) and HgBr_2_ ions [32, 33]. The hole transport layer is obtained from Ag_2_Te NCs that are cation exchanged using HgCl_2_ to form an Ag‐doped HgTe layer and then ligand exchange using ethanedithiol (EDT). Finally, a gold top contact is used for hole extraction. The IV characteristic of the diode is given in Figure 1e, showing a clear rectification that reaches 2.5 orders of magnitude. The dark current under reverse bias is at around 0.1 mA cm^−2^ under −0.5 V, while the *V*_OC_ is 220 mV under illumination by a 4 mW laser diode at 1.55 µm. Keeping the exact same procedure, just replacing the HgTe core by their core–shell counterpart, the I‐V curve appears strongly affected, with an enhanced rectification. The reverse dark current is reduced by two orders of magnitude now at 2 × 10^−6^ A cm^−2^, and the *V*_OC_ is increased to 370 mV. The only drawback is a loss in the photocurrent by a factor 7, which we attribute to the shell acting as a barrier that reduces the mobility. However, the dark current is reduced even further, so that overall, the light‐induced current modulation is increased by a factor 14.

**FIGURE 2:**
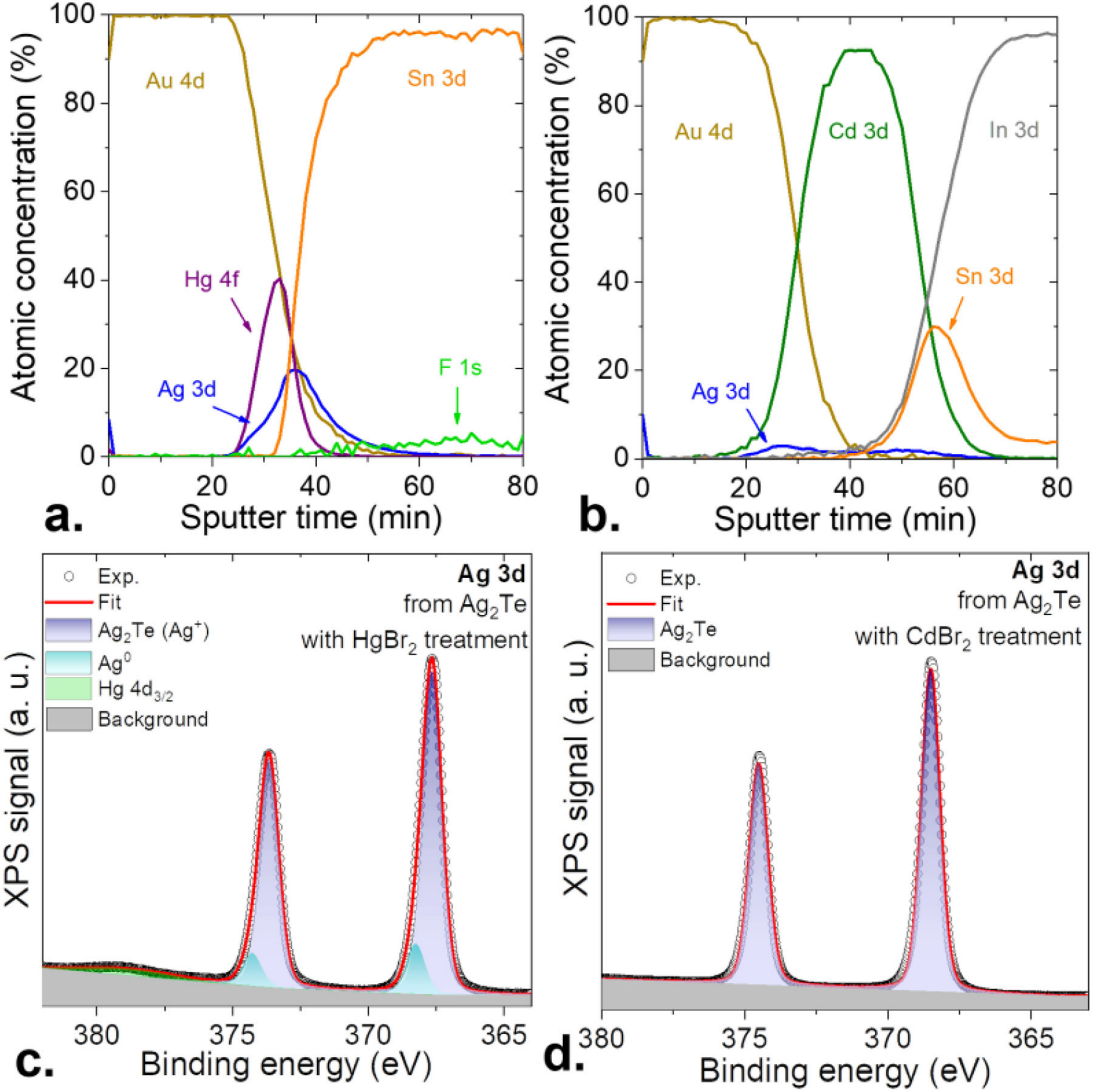
Structure/electronic‐properties correlation for the HgTe NC‐based diode stack. (a) (resp. (b)) Atomic ratio as a function of the Ar ion sputtering time for the conventional HgTe core NC stack (resp. for HgTe/CdS NC‐based optimized stack). Time zero corresponds to the top gold contact of the diode. Note that for the core–shell structure we track the Cd, because the Hg signal is weakene due to the presence of the shell (c) (resp. (d)) Ag 3d core level from Ag_2_Te NC film treated with HgBr_2_ (resp. treated with CdBr_2_).

Encouraged by the increase in *V*_OC_ and the drop of the dark current, we further optimize the surface chemistry of the involved NCs. For HgTe/CdS, the ink is now prepared using CdCl_2,_ replacing the HgCl_2_, while for the Ag_2_Te layer, the HgBr_2_ treatment is replaced by a CdBr_2_ processing, see method section for a full description of the procedure. By doing so, not only the surface passivation is improved, but also the Ag_2_Te layer starts to transform into (Ag‐doped) CdTe. The *p* character of this layer and its wider bandgap nature compared to HgTe, make it acts as a unipolar barrier [34, 35], preventing the flow of dark electrons (Figure S4). The second benefit of this process is highlighted in Figure 2c,d. As Ag_2_Te is cation‐exchanged with HgBr_2_, it tends to form metallic Ag (Figure 2c), which will later act as an exciton quencher. This process does not happen when the film is treated with CdBr_2_, as shown in Figure 2d.

To understand why the core/shell‐based diode stack leads to a higher rectification ratio, we performed in situ profiling of the diode using a combination of Ar ion sputtering to etch and X‐ray photoemission spectroscopy (XPS) analysis (Figure 2a,b). The depth profile for the conventional stack is displayed in Figure 2a. A striking observation is that interfaces are not sharp, highlighting a strong species interdiffusion. This is somehow expected due to the use of the cation exchange process, but the intermixing is further enhanced by the strong tendency of Hg to form amalgams [36]. This leads to gold composition leaking up to the SnO_2_ bottom layer, while silver is mostly located far from the gold electrode, where it was initially positioned. Though it does not prevent the diode operation, it is also certain that the intermixing of species prevents a fine control of the composition and, consequently of the doping landscape. The observation of such strong intermixing justifies a posteriori the performance improvement obtained by Lan [18], and Jeong's group [37], who replace the Hg processing of the Ag_2_Te layer by the use of Ag/Au‐doped HgTe NCs, for which the doping is done at the synthesis level. In the case of core–shell HgTe/CdS NCs, with the new surface chemistry, the interfaces are still not sharp, but the interdiffusion is drastically reduced (see Figure 2b). Gold and silver are now localized on the upper side of the absorbing layer, while the tin is located on the other side. The reduced interdiffusion enables a better‐defined *p‐i‐n* heterojunction and contributes to the reinforced diode character, electrically observed in Figure 1e.

**FIGURE 3:**
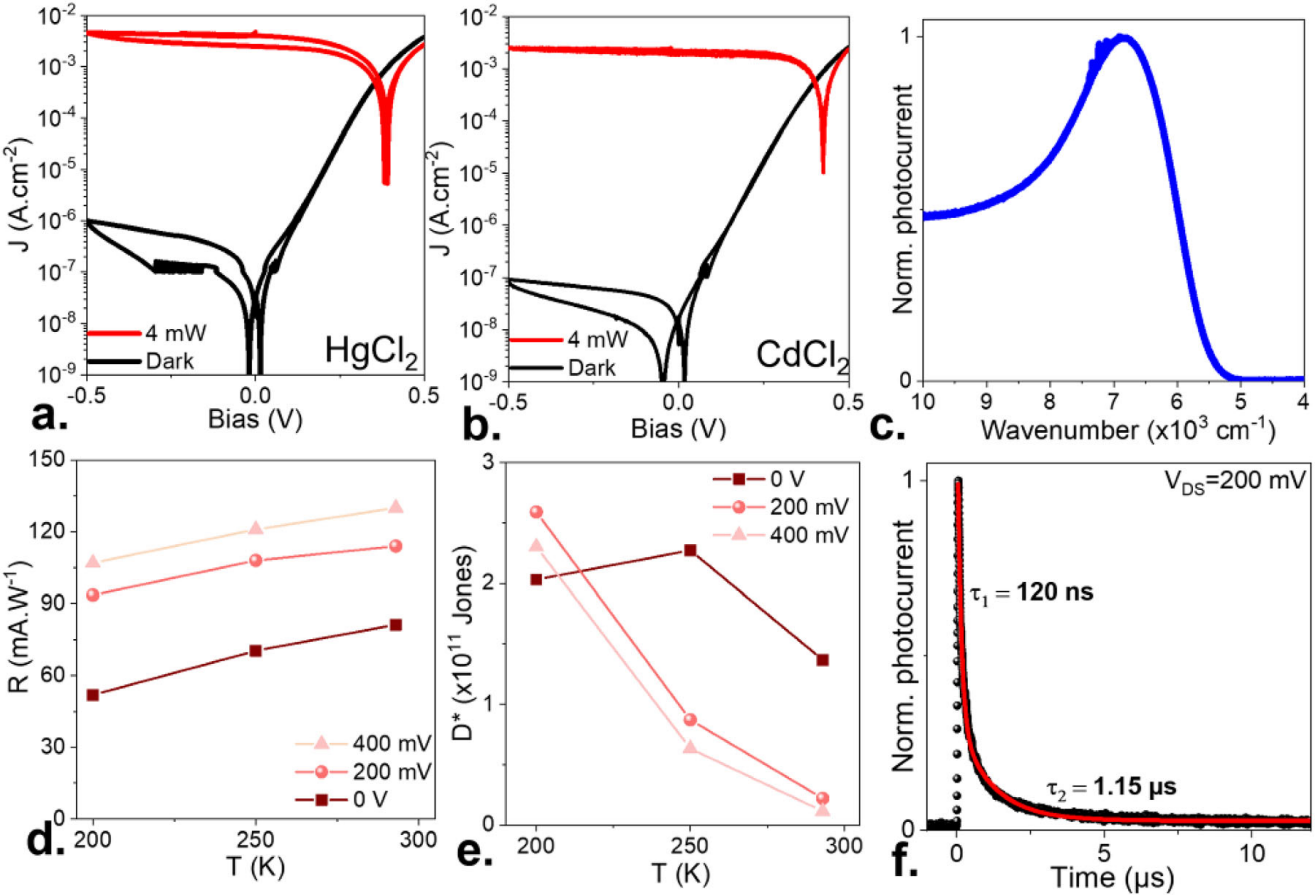
Photodiode with optimized surface passivation. (a) (resp (b)) Current density as a function of the applied bias in the dark and under illumination (1.55 µm–4 mW of power) for HgTe/CdS NCs, using HgCl_2_ (resp. CdCl_2_) as surface ligands (combined with mercaptoethanol). (c) Photocurrent spectral response acquired at room temperature under 0 V for HgTe/CdS NCs. (d) (resp (e)) Responsivity (resp specific detectivity) for the diode as a function of the operating temperature under various biases. (f) Photocurrent time response after illumination by a 1 ns long pulse at 1.57 µm. Data are collected while the pixel is biased under −200 mV and for a 0.15 mm^2^ pixel area. The red curve is obtained using a dual exponential decay, whose characteristics are provided on the graph.

The benefit of the optimized surface chemistry is highlighted in Figure 3a,b. The dark current further drops down to 10^−7^ A cm^−2^ at −0.5 V, and for room temperature operation, the lowest value among HgTe NCs‐based photodiode operating in the SWIR. The rectification now reaches four orders of magnitude, and the *V*_OC_ is as high as 420 mV. This value is almost twice that of the reference core NC‐based diode and, for the first time with HgTe‐based NCs exceeds *E*_G_/2. Also note that the fill factor of the photocurrent *J–V* curve is closer to one (Figure 3b) compared to the core‐only material (Figure 1e) and even compared to the core–shell with conventional HgCl_2_ treatment (Figure 3a). Away from the reverse mode, where the device is highly resistive and where hysteresis is due to capacitive effects, the *I–V* curve also displays a reduced hysteresis compared to the reference, suggesting a better management of the free ions. Thus, the optimized procedure suggests better surface passivation of the material.

We then quantify the performance of the resulting photodiode. At room temperature, the device presents a 5500 cm^−1^ cut off (1.8 µm), see Figure 3c. The responsivity, at room temperature, is at around 70 mA.W^−1^ under zero bias and increases to 100 mA.W^−1^ under −200 mV (Figure 3d). The time response of the diode to a 1 ns infrared pulse presents a fast decay component at around 120 ns, followed by a slower one in the ≈1.2 µs range, see Figure 3f.

Upon cooling, the photoresponse tends to drop slightly, typically by 10% as the device gets cooled down to 200 K. The noise spectral density [38] (see Figure S5) shows white noise under zero bias, consistent with the behavior observed for HgTe NCs resistive diodes, and then transforms into a frequency‐dependent noise under higher bias [39]. The associated specific detectivity at 0 V and room temperature reaches 1.5 × 10^11^ Jones (Figure 3e). This value matches the range of the best performing HgTe NC diodes, in spite of a modest response typically one order of magnitude lower that of the best device, as indicated in Table 1. This sets a clear objective for future improvement: managing the light‐matter interaction by coupling the diode to a photonic cavity that would recycle photons, thereby increasing device absorption. Overall, we observe an increasein detectivity upon cooling, except for the point at 200 K and 0 V, which appears setup‐limited, since the magnitude of the noise reaches the noise floor of the amplifier (≈5 fA.Hz^−1/2^). Under 200 mV and for 200 K operation, the specific detectivity [38], is now increased to 2.5 × 10^11^ Jones.

Complementary to the increase of *V*_OC_, we now aim for direct evidence on how the electronic structure gets affected by the presence of the shell. To do so, we use photoemission imaging as a method to directly track the remote doping induced by the transport layer onto the optically absorbing layer. Photoemission serves as a sensitive probe of the local environment, while the imaging allows for correlation between the diode structure and its environment.

**FIGURE 4:**
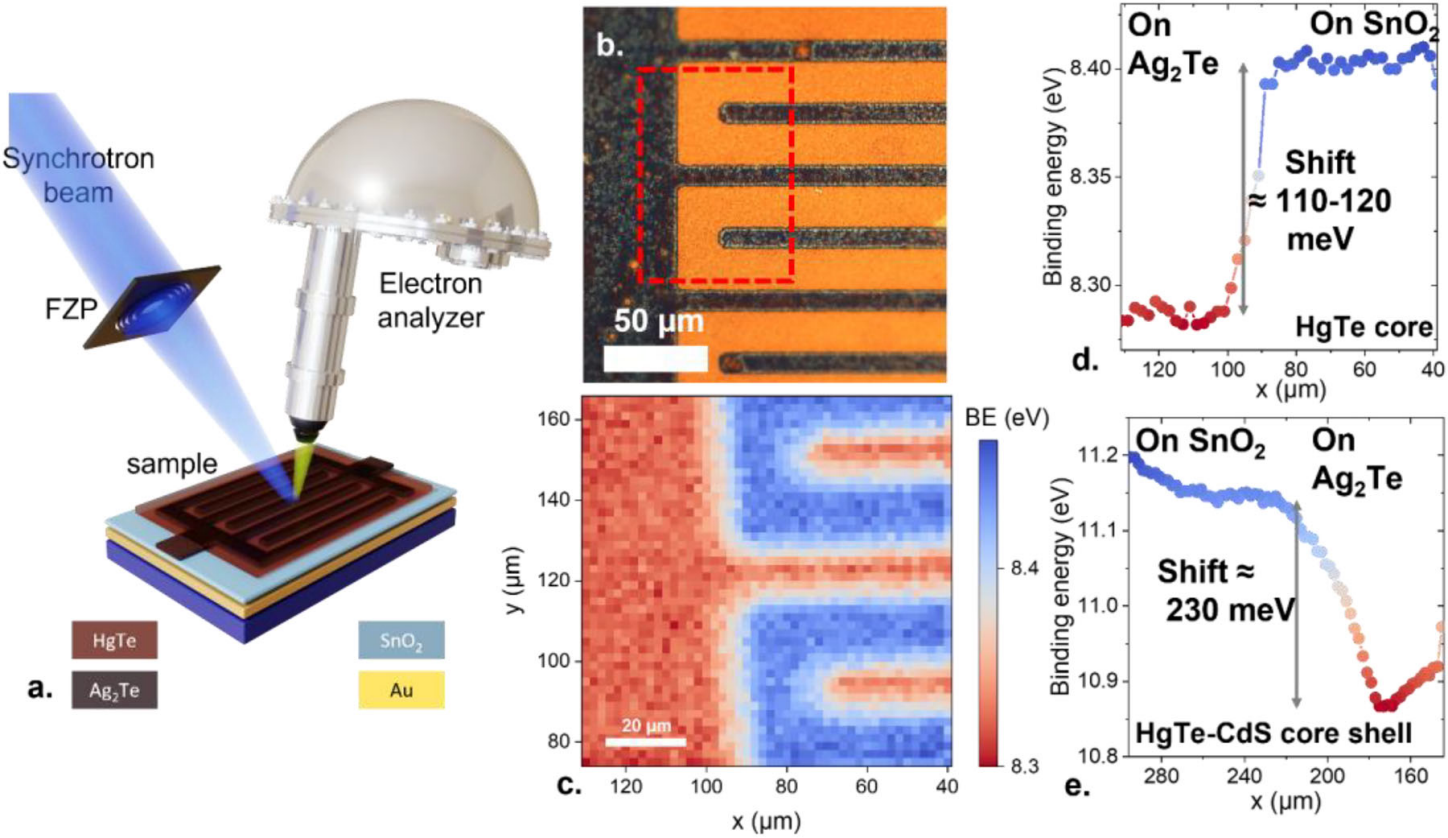
Direct imaging of the surface potential using X‐ray photoemission imaging. (a) Schematic of the X‐ray photoemission imaging setup. A 95 eV synchrotron photon beam is focused using a Fresnel zone plate (FZP), forming a 700 nm spot at the sample level. The energy of the ejected electron is then measured using an electron analyzer. The beam is scanned across the planar *p‐n* diode and, for each point, we collect the spectrum relative to the Hg 5d/Cd 4d state. (b) Optical image of the planar *p*‐*n* junction made of SnO_2_/Ag_2_Te heterojunction functionalized by HgTe NCs. (c) Binding energy map of the Hg 5d state over an area corresponding to the one highlighted with a red dashed line in part (b). (d) (resp. (e)) Profile of the Hg 5d from HgTe NCs (resp. Cd 4d from HgTe/CdS core–shell NCs) state as the beam is scanned from an Ag_2_Te‐coated area to an SnO_2_ area.

The method consists of shining a focused soft X‐ray beam onto the sample and collecting, for each point, a photoemission spectrum. In practice, a 95 eV photon beam is focused using a Fresnel zone plate, generating a 700 nm spot size at the sample level, see Figure 4a. The sample is scanned in the perpendicular plane, so that a hyper‐spectral map can be acquired [53, 54, 55, 56, 57].

The method raises two constraints on the sample level. First, soft X‐ray energy is being used to optimize the focusing through the Fresnel zone plate and, consequently, only low‐energy core levels can be tracked (i.e., with energy below 95 eV). In our case, several possibilities are available (Figure S6 for survey spectra) with Hg 5d (BE = 8.3 eV), Cd 4d (BE = 11.4 eV), and Te 4d (BE = 40.2 eV). The second requirement relates to the short escape depth of photoelectrons at this photon energy, typically below 1 nm. In such a condition, only the top surface of the sample is probed and therefore, the tracked layer must be the top surface. As the measure is not compatible with the buried character of the material within a diode stack, we prepare a planarized version of the diode [55, 57]. A silicon substrate is first coated with gold, this conductive layer will prevent charge accumulation and the resulting electrostatic shift of the photoemission feature. The substrate is then coated with SnO_2_. Then, using optical lithography, we pattern the resist and deposit Ag_2_Te within the pattern. After lift‐off, a textured substrate is obtained presenting SnO_2_ and Ag_2_Te top surface. Finally, HgTe core NCs or HgTe/CdS NCs are deposited as the top layer, see Figure 4 and Figure S7 for the fabrication process.

In the optical image (Figure 4b), Ag_2_Te with a narrow bandgap appears in black, while SnO_2_, which is transparent appears orange and enables seeing the gold underneath. An area corresponding to the one highlighted by the red dashed line in Figure 4b, is then mapped out using the photoemission imaging. For each point, we collect a spectrum relative to the targeted core level. We then fit all the spectra to determine their actual binding energy and propose a mapping of the zone based on the peak binding energy, see Figure 4c.

We observe a clear correlation between the patterning and its impact on the NC electronic structure. The material on SnO_2_ presents a systematically higher binding energy that the one on Ag_2_Te. This shift is consistent with the material being more *n‐type* once coupled to SnO_2_ and more *p* when over the Ag_2_Te. The second striking behavior is the difference of magnitude in the signal: for the core, the modulation over the *n–p* interface is about 110–120 meV (Figure 4d), whereas for the core–shell (Figure 4d,e), the modulation is approximately twice as large, reaching 230 meV. With this method, we directly measure a quantity proportional to the built‐in potential, although partially screened by the fact that only the top surface is probed using photoemission. We attribute the smaller built‐in potential shift compared to that observed for *V*_OC_ to the fact that the planar structure fabrication misses the formation of the CdTe barrier over one side of the diode.

Since the potential of this material for imaging has already been demonstrated [25], we rather focus here on its use for spectroscopic applications [58]. The low dark current from this diode enables longer integration times and thus longer averaging of the signal. We typically target the design of a photodiode with a narrow response, which can either be used for the detection of chemical species or to filter out photons that do not match spectrally with the targeted window. For instance, in LIDAR systems, only the photons scattered from objects illuminated by the source should be detected, whereas any other photon coming from an additional source (sun, laser, LED…) acts as a parasitic signal. Adding a filter is certainly the most straightforward strategy [59], however in this concept, the filtering only generates loss. Here, we rather target the coupling of the diode to a dielectric Bragg mirror, so that the diode is used as a detection medium within a cavity, therefore taking advantage of field enhancement in the optical resonator. As a proof of concept, we target the design of a detector enabling a narrow band at the telecom wavelength 1.55 µm or 6450 cm^−1^. Such a concept of dielectric cavity is inspired by vertical cavity surface emitting laser (VCSEL) design and has already been reported as a strategy to boost the luminescence of NC films, possibly up to lasing [60], shape their spectra [61, 62], or even to generate polaritons [63]. However, few works have been dedicated to the integration of a detector within the cavity [64, 65]. Compared to pure optical pumping, the integration of a diode requires managing the optical and electrical design simultaneously.

**FIGURE 5:**
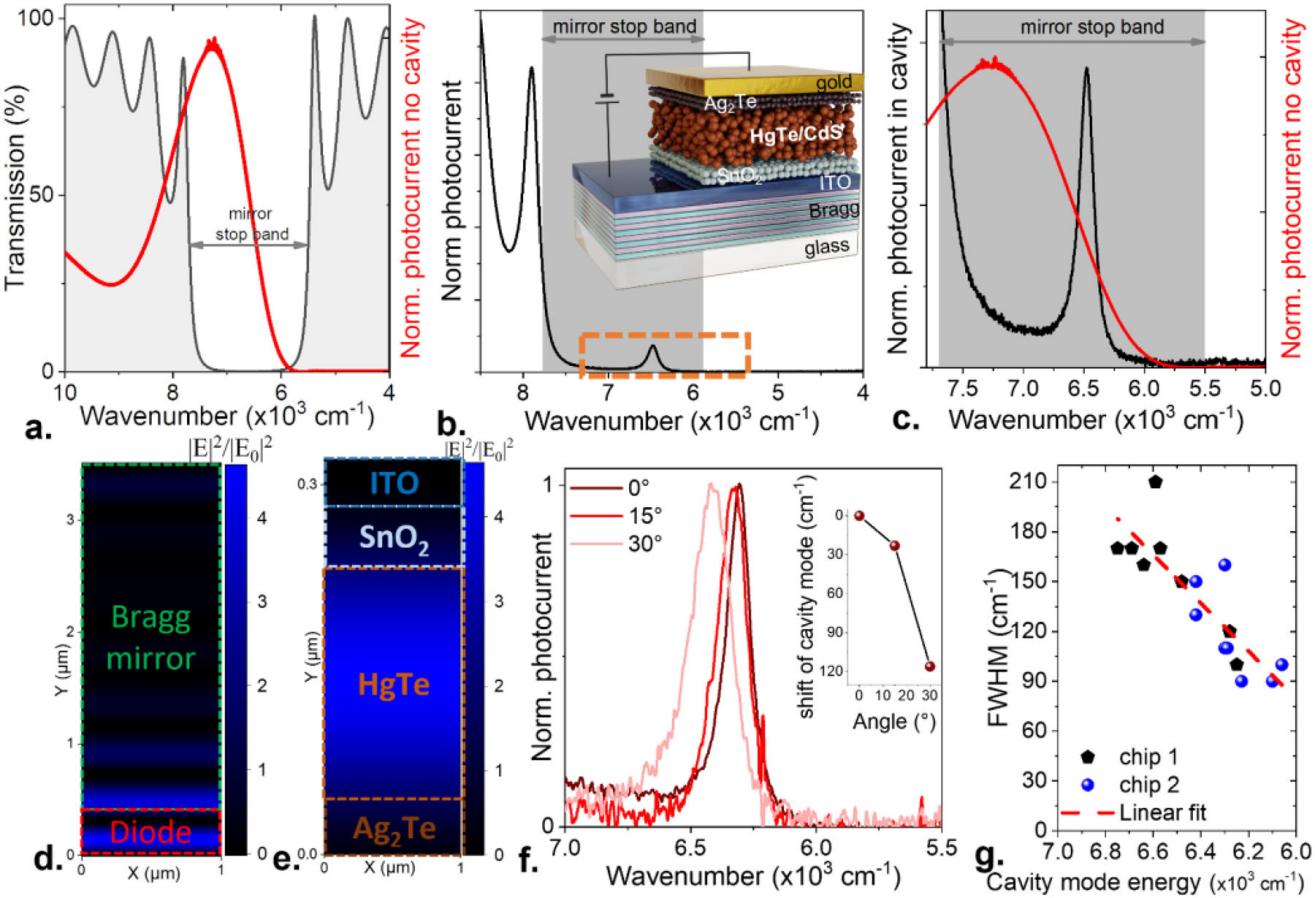
Integration of the photodiode into a dielectric cavity for narrow detection. (a) Transmission of the periodic Bragg mirror and photocurrent spectra of the integrated material in the absence of cavity effect. (b) Photocurrent spectra of the diode coupled to the cavity. The area highlighted by an orange dashed line is zoomed in part (c). The inset is a schematic of the diode stack coupled to the dielectric mirror. (c). Comparison of the diode response inside and outside the cavity. (d) Simulated map of the square of the field normalized by its incident value for the diode coupled to the Bragg mirror. Light illumination comes from the top. (e) Zoom‐in on the graph from part d in the area corresponding to the diode stack. (f) Photocurrent spectra for the cavity mode for three incidence angles of the detector. The inset shows the shift of the cavity mode energy as a function of the incident angle to the normal of the sample. (g) FWHM of the cavity mode as a function of the cavity mode energy for two chips containing eight diodes each. A linear fit of the trend is also proposed.

To target narrow optical features, a dielectric cavity, based on low‐loss materials, appears better suited than a plasmonic strategy [66] since the linewidth of the latter is broadened by intrinsic metal losses. TheBragg mirror (Figures S8 and S10) is made of 15 layers alternating SiO_2_ as low‐index medium and TiO_2_ as highindex medium. Each layer is made to have a thickness of around *λ/4n* with *n* the refractive index of the layer, so that the total thickness is 3.1 µm. The transmission of the mirror is displayed in Figure 5a with a stop band between 7500 and 5500 cm^−1^. To generate narrow features, we also slightly update the absorbing material so that the mirror stop‐band overlaps with the tail of the absorption rather than being spectrally resonant with the excitonic feature. The photocurrent of the material is shown in Figure 5a and now presents a cut‐off at around 6000 cm^−1^ (as opposed to 5300 cm^−1^ for the other devices presented in the text). The diode is then built following a conventional procedure as the one previously described. In this architecture, the top gold layer has a dual role of being the photohole extractor and a mirror that closes the cavity, see a schematic as the inset of Figure 5b. We have verified that the diode behavior and low dark current are maintained while the substrate is changed (Figure S9 for *I–V* curve).

For a diode with suitable thickness, typically in the 180–200 nm range, we see a cavity mode appearing in the mirror stop band, see Figure 5b. To better see the benefit of the cavity in the narrowing of the photoresponse, we have superimposed the response of the conventional diode that displays a 1700 cm^−1^ FWHM with that of the cavity, for which the linewidth is typically 130 ± 40 cm^−1^ (90 cm^−1^ for the narrowest device), corresponding to a factor 13 in the linewidth decrease, see Figure 5c. It is moreover worth noticing that the filtering action of the cavity also comes with field enhancement, which reaches a factor 4.5, as shown by the simulations in Figure 5d,e. Though narrow, the response presents some degree of tunability thanks to the dispersion of the cavity, see Figure S11 and Figure 5f. A spectral shift of the order of the linewidth can be obtained through the rotation of the device by 30° (Figure S11c)

To finish, we would like to discuss the fundamental limit of the linewidth to determine if this value of ≈100 cm^−1^ can be further narrowed [59, 65]. Bossavit et al., for example reported a cavity mode in photoconductive NC‐based device as narrow as 30 cm^−1^ [65]. However, their photoconductive devices present a dark current orders of magnitude larger, and their narrow linewidth was obtained at the price of a mostly empty cavity and therefore having weak absorption. To determine how narrow the response of a diode coupled to a cavity can be made, we have measured the linewidth of the cavity mode as a function of the device area (Figure S12) and found no correlation neither on photocurrent (Figure S12a) nor on optical reflectivity (Figure S12b), while the equivalent pixel/spot size is tuned over one order of magnitude. Indeed, in the case where two Bragg mirrors are facing each other, their parallelism is a critical parameter [65]. The presence of a tilt angle causes different cavity thicknesses to be integrated over the device area, which broadens the cavity mode. To overcome this drawback, the pixel size must be reduced to prevent the response from being averaged over a wide range of thicknesses. Here, the geometry where gold is evaporated over the device leads to a more conformal deposition and thus the fabrication process is self‐aligning. This can be leveraged to design a large pixel that therefore collects more light.

Conversely, we observe a clear correlation between the cavity linewidth and its energy as shown in Figure 5g. Given that smaller energy (i.e., smaller wavenumber) cavity modes are closer to the material cut‐off and thus present a reduced absorption, this suggests that a significant fraction of the cavity linewidth may be inherent to its absorption. To address this question, we conduct a series of simulations (Figure S13), as the extinction coefficient (*k*) of the infrared absorbing layer (i.e., the HgTe/CdS together with the Hg cation exchanged Ag_2_Te layer) is artificially modulated. As *k* is decreased, the cavity mode gets narrower and stronger in magnitude. The FWHM drops from 270 to 23 cm^−1^ as *k* is tuned from the band edge value (*k* = 0.1) to 10^−3^ [67]. The experimental value (90–200 cm^−1^ range) is found to be smaller than the band edge value due to the intentional detuning of the band edge with the cavity, as stressed in Figure 5a. Potentially, the cavity mode could be made as narrow as ≈20–30 cm^−1^, but this will come at the price of reduced absorption given the fact that the integrated area of the cavity mode appears almost unaffected by the *k* value tuning (Figure S13).

To summarize, we have shown that the performance limitations of HgTe‐based NC diodes can be overcome through targeted surface‐chemical engineering and photonic integration. The introduction of an ultrathin CdS shell, together with Cd‐based cation‐exchange chemistry in both the absorber and hole‐transport layers: (*i*) yields significantly improved passivation of surface states, (*ii*) reduces species interdiffusion, enabling a clearer *p‐i‐n* doping profile, and (*iii*) forms a unipolar barrier. This strategy produces a two‐order‐of‐magnitude reduction in dark current and elevates *V*_OC_ to 420 mV, marking the first report of a HgTe NC diode surpassing the *E*_G_/2 threshold. Complementary photoemission microscopy directly reveals the enhanced built‐in potential enabled by the shell, confirming the microscopic origin of the improved diode characteristics. Building on the resulting low noise and high rectification, we demonstrate the integration of these NC diodes within a dielectric Bragg cavity to realize narrowband infrared detection that maintains a spectral tunability through angular dispersion of the absorption. The cavity–diode coupling produces spectral linewidths as small as 90 cm^−^^1^ near 1.55 µm. This value is fully driven by the diode absorption, and further narrowing would require to also reduce the device responsivity. This architecture simultaneously enhances spectral selectivity and preserves electrical responsivity, offering a compact alternative to external optical filters. Together, these advances position core/shell HgTe NCs as a robust platform for next‐generation infrared photodetectors, with strong potential in spectroscopy, LiDAR, and low‐background imaging.

## 3 Methods

### 3.1 Chemicals

Mercury chloride (HgCl_2_, Alfa Aesar), mercury bromide (HgBr_2_, Alfa Aesar), tellurium powder (Te, Alfa Aesar, 99.99%), cadmium nitrate tetrahydrate (Cd(NO_3_)_2_.4H_2_O, Alfa Aesar, 99,9%), cadmium chloride anhydrous (CdCl_2_, Thermo Scientific, 99%), cadmium bromide (CdBr_2_, Alfa Aeasar, 98%), carbon disulfide (CS_2_, Merck, >99,9%), trioctylphosphine (TOP, thermofisher scientific, 90%), oleylamine (OLA, Acros, 80%–90%), octadecene (ODE, Acros Organics, 90%), dodecanethiol (DDT, Sigma–Aldrich, 98%), oleic acid (99%, Alfa Aesar), ammonia solution 30% vol. (NH_4_OH, Carlo Erba), aniline (Merck, >99,5%), methanol (MeOH, Carlo Erba, 99.8%), acetone (VWR), absolute ethanol (EtOH, VWR), isopropanol (IPA, VWR), toluene (Carlo Erba, 99.3%), N,N‐dimethylformamide (DMF, VWR), 2‐mercaptoethanol (MPOH, Merck, >99%),. All chemicals are used as received, except oleylamine, which is centrifuged before use. **Mercury compounds are highly toxic. Handle them with special care**.

### 3.2 1 M TOP:Te Precursor

Te powder (6.35 g) was mixed in 50 mL of TOP in a three‐neck flask. The flask was kept under vacuum at room temperature for 5 min and then the temperature was raised to 100°C. Furthermore, degassing of the flask was conducted for the next 20 min. The atmosphere was switched to Ar and the temperature was raised to 275°C. The solution was stirred until a clear orange coloration was obtained. The flask was cooled down to room temperature and the color switched to yellow. Finally, this solution was transferred to a nitrogen‐filled glove box for storage.

### 3.3 Core‐Only HgTe NCs

In a 100 mL three neck flask, 540 mg of HgCl_2_ (2 mmol) and 50 mL of oleylamine are degassed under vacuum at 110°C for 30 min. Then, the atmosphere is switched to N_2,_ and the temperature stabilized at 58°C. Meanwhile, 2 mL of TOP:Te (1 M) are extracted from the glove box and mixed with 8 mL of oleylamine. Then, the TOP:Te solution is quickly injected. After 3 min, 10 mL of a mixture of 10% DDT in toluene are injected and a water bath is used to quickly decrease the temperature. The content of the flask is split over 3 centrifuge tubes. The solution is precipitated first with MeOH. After centrifugation, the formed pellets are redispersed in one centrifuge tube with toluene. The solution is precipitated a second time with absolute EtOH and sonicated for 3 min. Again, the formed pellet is redispersed in toluene. Then, the NCs are centrifuged in pure toluene to get rid of the lamellar phase. The solid phase is discarded.

### 3.4 Ink From HgTe NCs Core

Before deposition, the NCs are prepared into a concentrated ink. A volume of 0.65/OD_500_ (optical density taken at 400 nm diluted by a factor of 500) of the prepared HgTe solution, 0.2 mL of DMF and 0.8 mL of exchange solution (30 mg HgCl_2_ and 2 mL of MPOH in 18 mL of DMF) are added to a centrifuge tube and agitated thoroughly with vortex mixing and sonication. 5 mL of hexane is added to wash the solution, agitated thoroughly without sonication, then discarded after phase separation. The QDs were then precipitated by adding 4 mL of toluene and centrifugating at 6000 rpm for 3 min. The remaining pellet was dried for 60 s under vacuum, then redispersed in 250 µL of DMF by agitating and sonicating.

### 3.5 NC Film Preparation

The NCs are then deposited on the various substrates prepared before. A few drops of filtered DMF were deposited on the substrate and left for 30 s, then dried by spincoating at 4000 rpm for 30 s. Then, 15 µL of previously prepared ink is deposited on the substrate and spincoated at 1250 rpm for 180 s then 6000 rpm for 60 s. The contact pads are then cleaned using a cotton swab.

### 3.6 Ammonium Phenyldithiocarbamate Synthesis (NH_4_PDTC)

In a 2‐neck 100 mL flask under nitrogen bubbling at room temperature, 25 mL of NH_4_OH, 25 mL acetone and aniline (10 mL, 0.11 mol) are introduced and cooled down with an ice bath. Then, CS_2_ (12 mL, 0.2 mol) is added dropwise with vigorous stirring. The solution turned red after 5 min, and a pale‐yellow crystalline product slowly precipitated over the following 90 min. The product is collected by vacuum filtration and washed four times with cold absolute ethanol. The NH_4_PDTC is dried under vacuum and used directly to prevent decomposition.

### 3.7 Cadmium Bis(phenyldithiocarbamate) Synthesis [Cd(PDTC)_2_] 

In an Erlenmeyer flask, NH_4_PDTC (600 mg, 3.2 mmol) was dissolved in 50 mL of water. The solution was clear yellow. Then, Cd(NO_3_)_2_.4H_2_O (493 mg, 1.6 mmol) was dissolved in 40 mL water and added dropwise to the flask over 5 min, leading to immediate precipitation of Cd(PDTC)_2_ as a pale yellow powder. The product was collected by centrifugation, and washed with 25 mL of absolute ethanol. The precipitate was ground with a mortar and pestle, dried under a Schlenk line and stored in the freezer (Mw = 449 g mol^−1^).

### 3.8 HgTe Core for Core–Shell Material

In a 100 mL three‐neck flask, 36 mL of oleylamine was degassed under vacuum at 110°C for 1 h. Then the atmosphere was switched to N_2_ and the temperature was set at 90°C. Meanwhile, in a 20 mL vial, 194 mg (0.5 mmol) of HgBr_2_ with 256 µL of oleic acid (0.8 mmol) was dissolved in 3.6 mL of oleylamine under sonication and degassed at 110°C for 30 min. Then, the solution in the vial was cooled down to room temperature and switched to N_2_ atmosphere. 0.4 mL of TOP:Te (1 M) was injected into the vial. The solution turned orange quickly and was injected into the hot oleylamine in flask through a syringe. After 30 s, the reaction was quenched with 2 mL of a solution of DDT in toluene (10% v/v) and ice bath. The content of the flask was transferred to a falcon and precipitated by adding MeOH. The pellet was redispersed in toluene. The NCs were precipitated a second time with absolute EtOH and redispersed in toluene. The toluene solution was centrifugated to remove the unstable phase and then filtered with 0.2 µm PTFE filter. This material is the one used for the detection side.

### 3.9 CdS Shell Growth

CdCl_2_ (36 mg) was added to 36 mL of a 50% (v/v) oleylamine‐octadecene solution in a flask and was degassed at 110°C for 30 min. The mixture was then cooled down to 40°C and switched to N_2_ atmosphere. 7 mL of HgTe core solution (0.45 OD at 400 nm after a 500x dilution) was introduced to the flask, and 3 cycles of vacuum/N_2_ cycles were conducted. Cd(PDTC)_2_ (100 mg, 2 mmol) in 2.6 mL of a 25% (v/v) oleylamine‐octadecene mixture was then quickly injected at 40°C, and the flask was heated up to 80°C for 2 min. Then, degassing was done to remove CS_2_. The reaction was quenched by injecting 2 mL of a 10% (v/v) DDT/OA‐toluene solution and cooling down to room temperature. The content in the flask was transferred to a centrifuge tube and precipitated with EtOH. The pellet was redispersed in toluene and then precipitated again with IPA. After redispersion, the toluene solution was centrifugated to remove the unstable phase (the supernatant was kept).

### 3.10 Ink Preparation for Core–Shell

Before deposition, the NCs are prepared into a concentrated ink. A volume of 1.35/OD_500_ of the prepared HgTe‐CdS solution and 1 mL of exchange solution (20 mg CdCl_2_ and 2 mL of MPOH in 18 mL of DMF) were added to a centrifuge tube and agitated thoroughly with vortex mixing and sonication. 8 mL of hexane was added to wash the solution, agitated thoroughly without sonication, then discarded after phase separation. This step was repeated two more times. The QDs were then precipitated by adding 10 mL of toluene and centrifugating at 6000 rpm for 4 min. The remaining pellet was dried for 600 s under vacuum, then redispersed in 350 µL of DMF by agitating and sonicating.

### 3.11 SnO_2_

The pristine solution (SnO_2_‐P) is obtained by diluting 400 µL of commercial solution with 0.8 mL of distilled water. For lightly doped SnO_2_ (SnO_2_‐L), we dilute 200 µL of commercial solution with 1 mL of 24 mm NH_4_Cl solution in water. For heavily doped SnO_2_ (SnO_2_‐H), we dilute 200 µL of commercial solution with 1 mL of 48 mm NH_4_Cl solution in water.

### 3.12 Ag_2_Te CQD Synthesis

In a 50 mL three neck flask, 170 mg of AgNO_3_ is mixed together with 25 mL of oleylamine and 2.5 mL of oleic acid. The flask is then degassed under vacuum at room temperature for 15 min and then at 70°C for 10 min. The atmosphere is replaced to nitrogen and 2.5 mL of TOP is added into the solution. Then, the temperature of the flask is raised to 160°C. After 30 min, the solution becomes orange. At this step, 0.5 mL of TOP:Te (1 M) is injected and the reaction is quenched after 10 min with a water bath. The crude solution was stored in a freezer at −20°C. When needed, 800 µL of the unfrozen crude solution is precipitated with MeOH. After centrifugation, the formed pellet is redispersed in a mixture containing 200 µL DDT and 600 µL of chlorobenzene. Then, the NCs are precipitated by addition of methanol. After centrifugation the pellet is dispersed using 800 µL of chlorobenzene. Again, the NCs are precipitated by addition of MeOH and centrifugation. The pellet is this time dispersed in 1.6 mL of a (9:1 volume ratio) mixture of hexane:octane.

### 3.13 ITO Patterning

A glass slide substrate (1.1 mm thickness) was cleaned via sonication in acetone. The glass was rinsed with acetone, then isopropanol, and dried with a N_2_ gun. A further cleaning was made using an O_2_ plasma for 5 min. An adhesion promoter (TI PRIME) was spin‐coated onto the glass substrate and baked at 110°C for 120 s. Photoresist (AZ5214E) was spin‐coated and baked at 110°C for 60 s. The substrate was exposed to UV through a pattern mask for 1.5 s. The film was then baked at 110°C for 2 min to invert the resist. Then, a 40 s UV flood exposure was performed. The resist was developed using a bath of AZ726MIF for 25 s, rinsed in pure water, and finally dried with N_2_. After a short cleaning with O_2_ plasma, 50 nm of ITO was deposited by sputtering. The final lift‐off was performed by dipping the film in acetone for 30 min. The patterned substrate was rinsed using isopropanol and dried with an N_2_ flow.

### 3.14 Diode with ITO/Doped:SnO_2_/HgTe/Ag_2_Te/Au Stack

The ITO‐coated glass substrates (see above procedure used to define patterns) were sequentially cleaned in acetone and isopropanol. The substrates were exposed to ozone plasma for 5 mins. The SnO_2_‐L solution was deposited onto the patterned ITO substrate via spin‐coating. The SnO_2_‐H solution was deposited onto the SnO_2_‐L layer. The film was annealed on a hot plate at 70°C for 1 h. The HgTe NC ink was deposited onto the SnO_2_ film via spin coating. The thickness of the film was tuned with spin coating speed and ink concentration in DMF solvent. On top of HgTe ink film, the Ag_2_Te NC layer was spin‐coated at 2000 rpm. This layer is then cation‐exchanged, to do so we performed a HgBr_2_ treatment. 50 µL of HgBr_2_ diluted in methanol (10 mm) is dropped onto Ag_2_Te film and spin‐dried after 15 s. Then the film is rinsed using isopropanol. This procedure is repeated one more time. Finally, an ethanedithiol ligand‐exchange is performed by dipping the film in a solution made of 1% ethanedithiol in acetonitrile for 30 s. Then, the film was rinsed with fresh acetonitrile. A top gold (80 nm) electrode is deposited with thermal evaporation under a vacuum of ≈5 × 10^−6^ mbar with a rate of 3 Å/s. To ensure a high degree of homogeneity, the substrate holder is spinned during the deposition.

### 3.15 Diode With Core–Shell

The ITO‐coated glass substrates (see above procedure used to define patterns) were sequentially cleaned in acetone and isopropanol. The substrates were exposed to ozone plasma for 5 mins. The SnO_2_‐L solution was deposited onto the patterned ITO substrate via spin‐coating. The SnO_2_‐H solution was deposited onto the SnO_2_‐L layer. These two SnO_2_ layers were 40 nm thick. The film was annealed on a hot plate at 70°C for 1 h. The HgTe/CdS NC ink was deposited onto the SnO_2_ film via spin coating. The thickness of the film is tuned with spin coating speed and ink concentration in DMF solvent, typically, this layer was 200 nm thick. On top of HgTe/CdS ink film, the Ag_2_Te NC layer was spin‐coated at 2000 rpm. This layer was then cation exchanged, to do so, we performed a CdBr_2_ treatment. 50 µL of CdBr_2_ diluted in methanol (50 mm) was dropped onto Ag_2_Te film and spin‐dried after 15 s, this layer was 30 nm thick. Then the film was rinsed using isopropanol. This procedure was repeated one more time. Finally, an ethanedithiol ligand‐exchange was performed by dipping the film in a solution made of 1% ethanedithiol in acetonitrile for 30 s. The film was then rinsed with fresh acetonitrile. A top gold (80 nm) electrode was deposited with thermal evaporation under a vacuum of ≈5 × 10^−6^ mbar with a rate of 3 Å/s. To ensure a high degree of homogeneity, the substrate holder was spinned during the deposition.

### 3.16 X‐Ray Photoemission Spectroscopy

XPS measurements of Ag_2_Te were performed using a PHI GENESIS setup equipped with a monochromatic Al Kα X‐ray source (hν = 1486.6 eV) with a spot size of 100 µm in a normal emission configuration at room temperature. Core‐level spectra were acquired with a pass energy of 27 eV. Peak decomposition is carried out by using pseudo‐Voigt doublet lineshapes (20% Lorentzian weight) and considering linear/Tougaard backgrounds. The zero‐binding energy reference, corresponding to the Fermi level, was calibrated using the leading edge of a clean Ag foil.

### 3.17 Photoemission Microscopy

The spatially resolved photoemission maps were measured at the ANTARES beamline of Synchrotron SOLEIL. We used linearly horizontally polarized photons with a photon energy of 95 eV and MBS A‐1 hemispherical electron analyzer [3]. All measurements were conducted at 250 K, with an energy resolution better than 30 meV and a spatial resolution as low as 700 nm along the pn junction (indicated as X‐direction in all figures). For all maps, angular dispersion of the analyzer (±15°) is neglected by integrating the spectra over all angles. The samples were mounted on the sample holder of ANTARES beamline and directly grounded to it via silver paste. The sample holder was then grounded through the manipulator. The acquired data were processed using Wavemetrics Igor Pro. In particular, the binding energy maps were obtained by fitting a Gaussian doublet to the measured spectra point‐by‐point and then plotting the binding energy value of the Hg 5d_5/2_ or Cd 4d_5/2_ contribution. To evaluate the energy shift, the photoemission spectra were averaged over a small number of points (5 or 10) in the regions of interest. The binding energy calibration was performed by measuring the Fermi edge of a gold substrate mounted on the same sample holder of the device.

### 3.18 Ar Ion Sputtering Depth Profiling

Diode depth profiling was performed by alternating XPS acquisitions with ion sputtering. Experiments were carried out on a PHI GENESIS setup equipped with a differentially pumped Ar^+^ ion gun. Sputtering was conducted using Ar^+^ ions at 1 keV over a 2 × 2 mm^2^ area, with an ion current of 500 nA. The UHV chamber base pressure was 3 × 10^−9^ mbar, while during sputtering was 7 × 10^−9^ mbar. XPS spectra were acquired using an Al Kα (1486.6 eV) monochromatic source with a 50 µm X‐ray beam spot and a pass energy of 140 eV. The analysis area is positioned at the centre of the sputtered region. During XPS acquisition, charge compensation was applied using the instrument's electron/ion flood source. Elemental quantification was carried out using the instrument's relative sensitivity factors.

### 3.19 Electromagnetic Simulation

Optical properties of the structure are simulated with COMSOL Multiphysics, 2D Frequency Domain *Interface*. Floquet periodicity is set for boundary conditions at the side edges. A periodic port to generate the incoming electromagnetic wave is placed on the substrate side. Perfectly matched layers are added to absorb outgoing waves and minimize possible nonphysical reflections due to limited mesh size. Physics‐controlled mesh is enabled with an extremely fine element size. The dissipated power per unit volume in the metals and NCs is computed as the surface integral within the layer of the local dissipation, the electric part of the field (emw.Qe) then divided by the incident power defined in the port. For example, the absorption spectrum in the NC film is:

$$Abs_{\mathrm{NC}}\;\left(\omega \right)=\frac{P_{\mathrm{NC}}\left(\omega \right)}{P_{\mathrm{incidence}}}=\frac{\int \;\;\;\int emw\cdot Qe\;dS}{P_{\mathrm{incidence}}}$$

For the glass substrate, the real and imaginary parts of the refractive indices are set to 1.41 and 0, respectively. For the gold layer, we use the dielectric function from Drude model ε(λ) = 1 − 1/((λ_p_/λ) ^2^+i(λ_p_/λ).γ), with λ the wavelength in meter and γ = = 0.0048 without unit [68]. For the NC film, the real partis taken from ellipsometry measurement [67], made on a HgTe 6k ink (fairly stable and around 2.4 over the range of interest) while the imaginary part is inferred from the absorption spectrum of the core–shell ink after renormalization of the excitonic peak at 0.1 [67].

### 3.20 Dielectric Mirror Deposition

The top side of the glass is coated with an optimized interferential filter made of 15 alternating titanium pentoxide (Ti_3_O_5_, Neyco, 99.99%) and silicon dioxide (SiO_2_, Neyco, 99.99%) thin layers, starting with Ti_3_O_5_, with a total thickness of 3.1 µm. These layers have been deposited by ion beam assisted e‐beam evaporation (IBAD MEB 800, Plassys). Ion assistance is provided by an End Hall ion gun (eH1000 source, Kaufman & Robinson KRI). The design, synthesis, and refinement of the custom coating have been performed theoretically with a commercial software (Essential MacLeod, Thin Film Center Inc.).

SiO_2_ and Ti_3_O_5_ (denoted as L and H for low and high refractive indices materials, respectively) have been chosen because of their transparency in the considered spectral ranges but also for their refractive index contrast (*n*_L_ = 1.453 ± 0.018 at λ = 1550 nm, whereas and *n*_H_ = 2.309 ± 0.080 at λ = 1550 nm).

Both Ti_3_O_5_ and SiO_2_ were evaporated at a rate of 0.25 nm·s^−1^. Starting materials are 1 to 3 mm pieces of Ti_3_O_5_ and SiO_2_ put in molybdenum liners. The background pressure in the chamber was 1.0 × 10^−7^ mbar, and the working pressure was ≈ 3.0 × 10^−4^ mbar with flows of 10 standard cubic centimeters per minute (sccm) of Ar and 10 sccm of O_2_ for the ion source, and 10 sccm of Ar for the keeper (plasma bridge neutralizer). Discharge currents were set to 1.6 and 2 A during Ti_3_O_5_ and SiO_2_ deposition steps, respectively. Under those conditions, the ion source discharge voltage was 150 V and 75 V during Ti_3_O_5_ and SiO_2_ deposition steps, respectively. The optical thicknesses were followed in real time using both a quartz microbalance and an in situ spectroscopic ellipsometer (M2000, J A  Woolam) at 60.5° of incidence.

The actual deposited stack for each layer of H(LH)^7^ starting from glass, was: 168.1 nm (Ti_3_O_5_)/266.8 nm (SiO_2_)/162.3 nm (Ti_3_O_5_)/264.1 nm (SiO_2_)/162.9 nm (Ti_3_O_5_)/268.7 nm (SiO_2_)/ 170.3 nm (Ti_3_O_5_)/266.2 nm (SiO_2_)/169.6 nm (Ti_3_O_5_)/267.6 nm (SiO_2_)/173.8 nm (Ti_3_O_5_)/259.1 nm (SiO_2_)/172.2 nm (Ti_3_O_5_)/259 nm (SiO_2_)/153.8 nm (Ti_3_O_5_).

### 3.21 DC Photoresponse Measurements

The DC photoresponse measurements were collected using a Keithley 2634b as both a source of bias and an amperemeter. A 1550 nm laser diode was used as the light source. To vary the intensity of the laser, the total incident power was first measured for different input currents using a PM100A Thorlabs power‐meter and a Ge detector. The photocurrent was then calculated as the difference between the dark current and the current under illumination.

### 3.22 Photocurrent Spectra Measurements

The spectral dependence of the photoresponse is measured with a Fisher IS50 FTIR (CaF_2_ beamsplitter, white light illumination) while the sample is biased with a Femto DLPCA 200 amplifier, which is also used for amplifying the photocurrent signal before sending the signal back into the FTIR for spectral analysis. The spectra are typically obtained in continuous scan mode with 4 cm^−1^ resolution and averaged at least 32 times.

### 3.23 Modulated Photoresponse Measurements and Responsivity

To obtain responsivity values, the modulated signal of a blackbody heated at 980^0^C is measured. The sample is placed around 37 cm away from the blackbody and the incoming flux is modulated by an optical chopper with frequency set to 99 Hz. A window filtering out all wavelengths shorter than λ_filter_ = 1 µm is placed between the source and the sample. The incident power is then calculated as:

$$\begin{array}{c} P\;\left(W\right)=\;\pi \cdot A_{d}\cdot sin^{2}\left(\frac{\theta }{2}\right)\cdot \cos\left(\varphi \right)\cdot \int_{\lambda_{\mathrm{filter}}}^{\lambda_{\mathrm{cut}-\mathrm{off}}}\frac{2hc^{2}}{\lambda^{5}}\cdot \\ \frac{1}{\exp\left(\frac{hc}{\lambda kT}\right)-1}d\lambda \end{array}$$

where 𝐴_𝑑_ is the area of the photodetector, 𝜃 is the field of view, 𝜑 is the incident angle (typically assumed to be 0^0^), h is the Planck constant, c is the speed of light, k is Boltzmann constant, T is the blackbody temperature equal to 980^0^C. 𝜆_𝑐𝑢𝑡‐𝑜𝑓𝑓_ is taken from the absorption edge of the material.

### 3.24 Noise Measurements and Detectivity

The noise spectral density was collected using a SR780 spectrum analyzer, while the device is biased with a Femto DLPCA 200 amplifier. Noise spectra were typically acquired over a frequency range from 10 Hz to 1 kHz. The noise current density spectrum was obtained after averaging 100 scans. The values of detectivity at 99 Hz are then computed according to $D^{*}=\frac{R\sqrt{A_{d}}}{S_{I}}$, where ℛ is the responsivity of the device, *A*_d_ is the device area, and 𝑆_𝐼_ is the measured noise current density.

### 3.25 Micro‐FTIR Measurements

Infrared reflectance maps are collected using a Nicolet RaptIR+ infrared microscope from Thermo Fisher Scientific. The sample is placed on a motorized XYZ stage. The sample is illuminated using the infrared source from a Nicolet IS50 FTIR, which has passed through the interferometer (KBr beamsplitter). The light is focused on the sample using a 15x IR Cassegrain objective and collected in the same way to measure reflectance. The collected interferogram is then measured by a MCT detector with an optical velocity of 1.89 cm/sec and a gain of 1. Maps are performed by moving the stage point by point in X and Y directions and collecting an IR spectrum for each point, with a 150 µm × 150 µm aperture for data collection. The data are collected with 16 cm^−1^ resolution and 8 sample scans for the average.

## Conflicts of Interest

The authors declare no conflicts of interest.

## Supporting information




**Supporting File**: adma73019‐sup‐0001‐SuppMat.docx.

## Data Availability

The data that support the findings of this study are available from the corresponding author upon reasonable request.
