# Supplementary material for: Surface Passivation of HgTe Nanocrystals Enabling EG/2 Open‐Circuit Voltage and Their Coupling to Dielectric Cavity for Narrow Detection
Source: Adv Mater. 2026 Apr 10;38(26):e73019. doi: 10.1002/adma.73019 (PMC13155297; doi:10.1002/adma.73019)
Supplement: Supplementary file 1 — Supporting File: adma73019‐sup‐0001‐SuppMat.docx. [file ADMA-38-e73019-s001.docx]

*Supporting information for*

**Surface Passivation of HgTe Nanocrystals enabling E_g_/2 Open-Circuit Voltage and their Coupling to Dielectric Cavity for Narrow Detection**

Albin Colle^1^, Clement Gureghian^1^, Dario Mastrippolito^1^, Mariarosa Cavallo^1^, Jiho Roh^1^, Marco Paye^1^, Tommaso Gemo^1,2^, Diogo Almeida^1^, Adrien Khalili^1^, Yoann Prado^1^, Xavier Lafosse^3^, Sandrine Ithurria^4^, Mathieu G. Silly^5^, Pavel Dudin^5^, James K. Utterback^1^, José Avila^5^, Debora Pierucci^1^, Emmanuel Lhuillier^1*^

^1^ Sorbonne Université, CNRS, Institut des NanoSciences de Paris, 4 place Jussieu, 75005 Paris, France.

^2^ LYNRED, Actipole - CS 10021, 364 route de Valence, 38113 Veurey-Voroize, France

^3^Centre de Nanosciences et de Nanotechnologies, CNRS, Université Paris-Saclay, C2N, Palaiseau 92110, France.

^4^Laboratoire de Physique et d’Etude des Matériaux, ESPCI, PSL Research University, Sorbonne Université, CNRS, 10 rue Vauquelin, 75005 Paris, France.

^5^ Synchrotron SOLEIL, L'Orme des Merisiers, Départementale 128, 91190 Saint-Aubin, France.

*To whom correspondence should be sent: [el@insp.upmc.fr](mailto:el@insp.upmc.fr)

**Table of content**

[1. State-of-the-art study for HgTe NC-based photodiode 2](#_Toc222740335)

[2. Material characterization 2](#_Toc222740336)

[3. Performance of the diode with optimized surface chemistry 6](#_Toc222740337)

[4. Device for photoemission imaging 7](#_Toc222740338)

[5. Integration of the diode on a dielectric mirror 8](#_Toc222740339)

[6. REFERENCES 11](#_Toc222740340)

# State-of-the-art study for HgTe NC-based photodiode


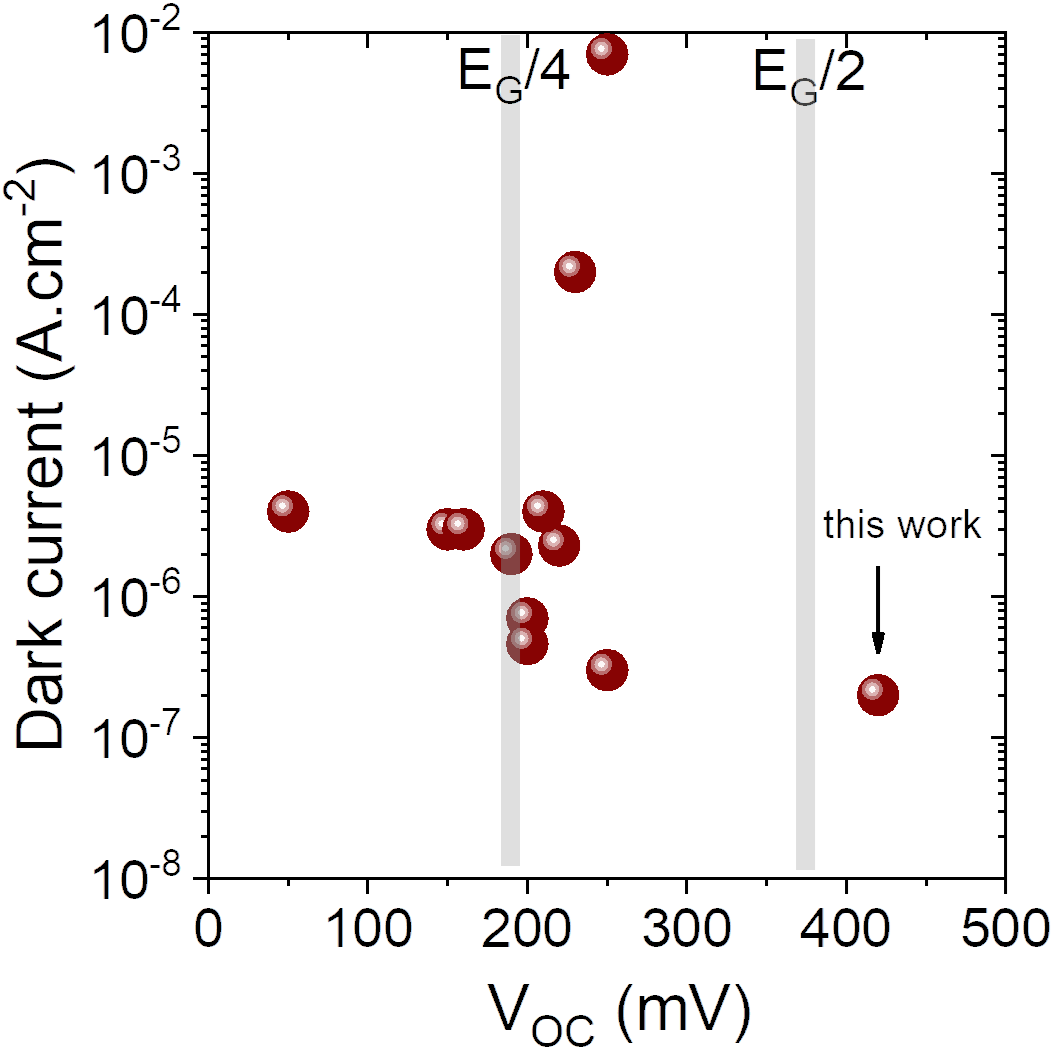


**Figure S 1 Dark current as a function of the open circuit voltage for HgTe NC-based diode.** Data are extracted from Table 1 in the main text.

# Material characterization

**Figure S 2** displays the absorption spectra from the core-only HgTe NCs (*i.e.,* tripod shape object as obtained from ref,^[1]^. Note that these are not the starting core for the core shell object, as requires spherical cores to favor even shell growth) and the HgTe-CdS material. CdS shell growth induces a redshift that we account for so that the final material presents a similar band edge at around 0.75 eV.


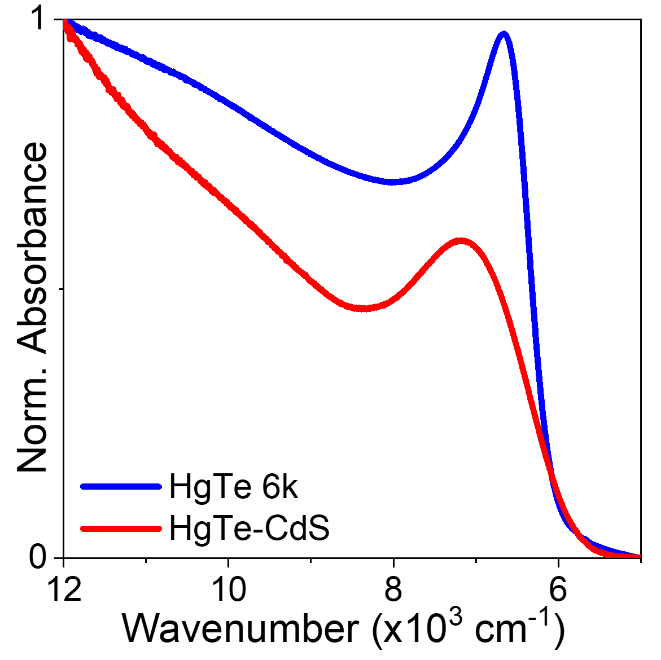


**Figure S 2 Absorption spectra for HgTe core and HgTe-CdS core shell.**

XPS analyses of the core and core-shell HgTe (/CdS) NCs have been conducted, as shown in **Figure S 3**. The core only material (**Figure S 3**a) displays an especially simple spectrum, with contributions of Hg, Te and C, the latter coming from the ligands (mercaptoethanol). It is worth stressing that there is no contribution from the O 1s state (expected at a 532 eV binding energy) in spite of air preparation of the sample and absence of an annealing step prior to measurement. This absence is in line with the previous report stressing the weak tendency of the material for oxidation.^[2]^

After shell growth (**Figure S 3**b), the contributions of Hg, Te, and C are completed by the one from Cd and S as expected. We also see a signature of the chlorine that corresponds to the complementary ligand (HgCl_2_ used together with mercaptoethanol).


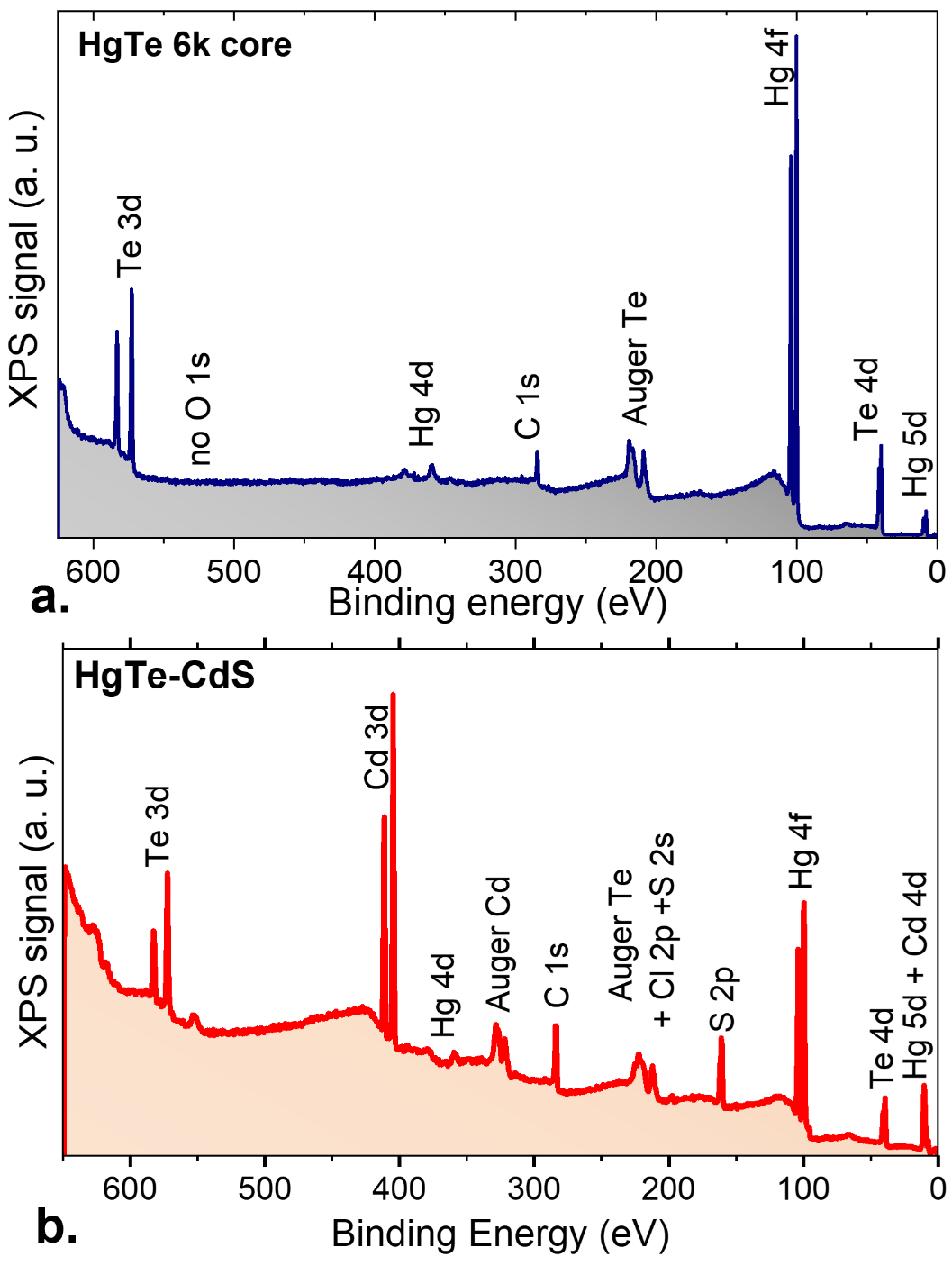


**Figure S 3 Survey photoemission spectra** acquired with a 700 eV photon energy for HgTe core (a.) and HgTe-CdS core shell (b.).

The most successful strategy to form a heterojunction with HgTe NCs, relies on the use of Ag_2_Te NCs which are further cation-exchanged with Hg to form an Ag-doped HgTe layer.^[3]^ In the updated procedure for core shell, this cation exchange procedure is replaced by CdBr_2_ treatment, rather forming Ag-doped CdTe. We measured that the energy of the valence band is barely affected, see **Figure S 4**, while the band gap of CdTe is much larger than that of HgTe layer for the same nanocrystal size, due to its larger bulk band gap and increased conduction effective mass. As a result, a large offset in the conduction band is generated and makes the layer act as a unipolar barrier.^[4]^


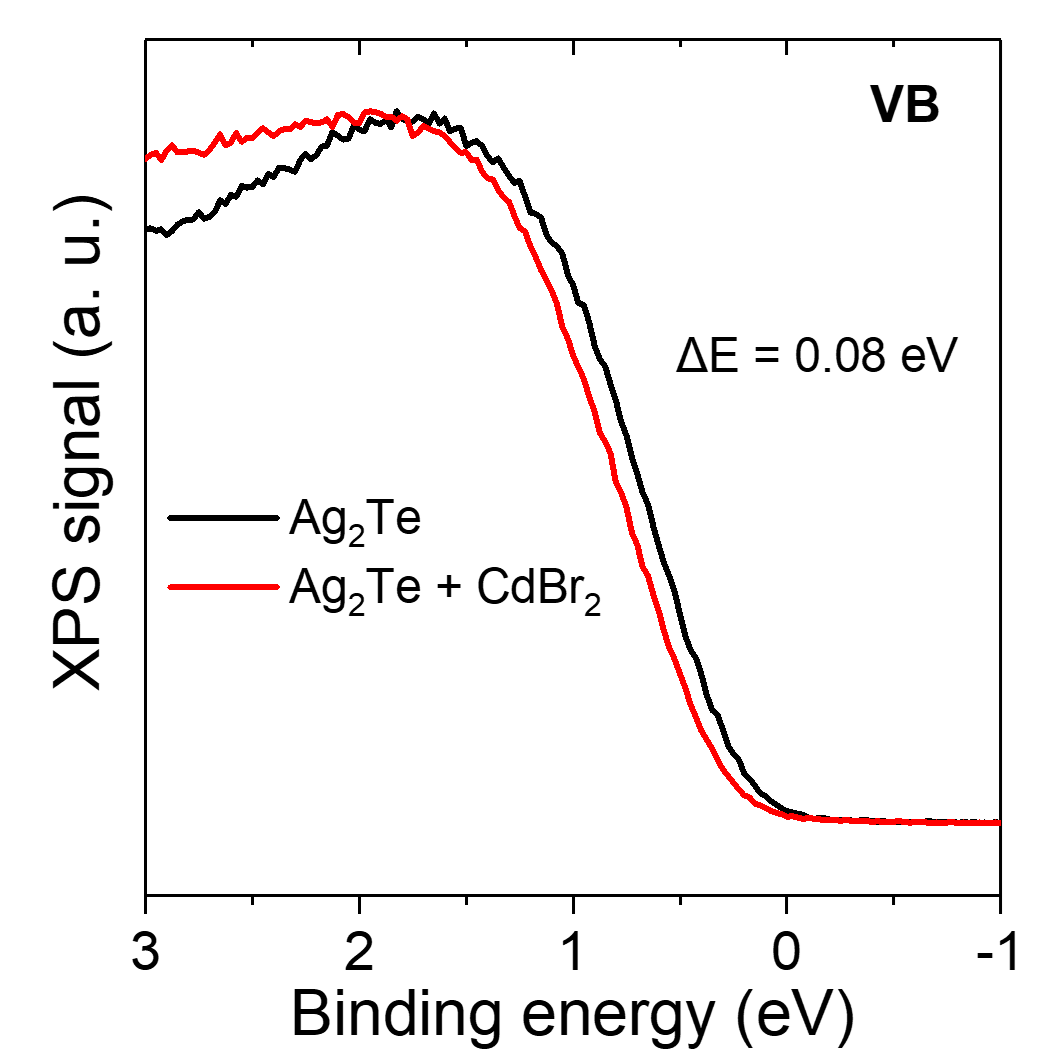


**Figure S 4 Impact of the CdBr_2_ treatment on the p-type layer**. Valence band signal for pristine Ag_2_Te and the one treated with CdBr_2._

# Performance of the diode with optimized surface chemistry

Noise in the diode under investigation comes under a white noise from at 0 V, and turns frequency dependent (1/f like),^[5]^ as bias is increased.


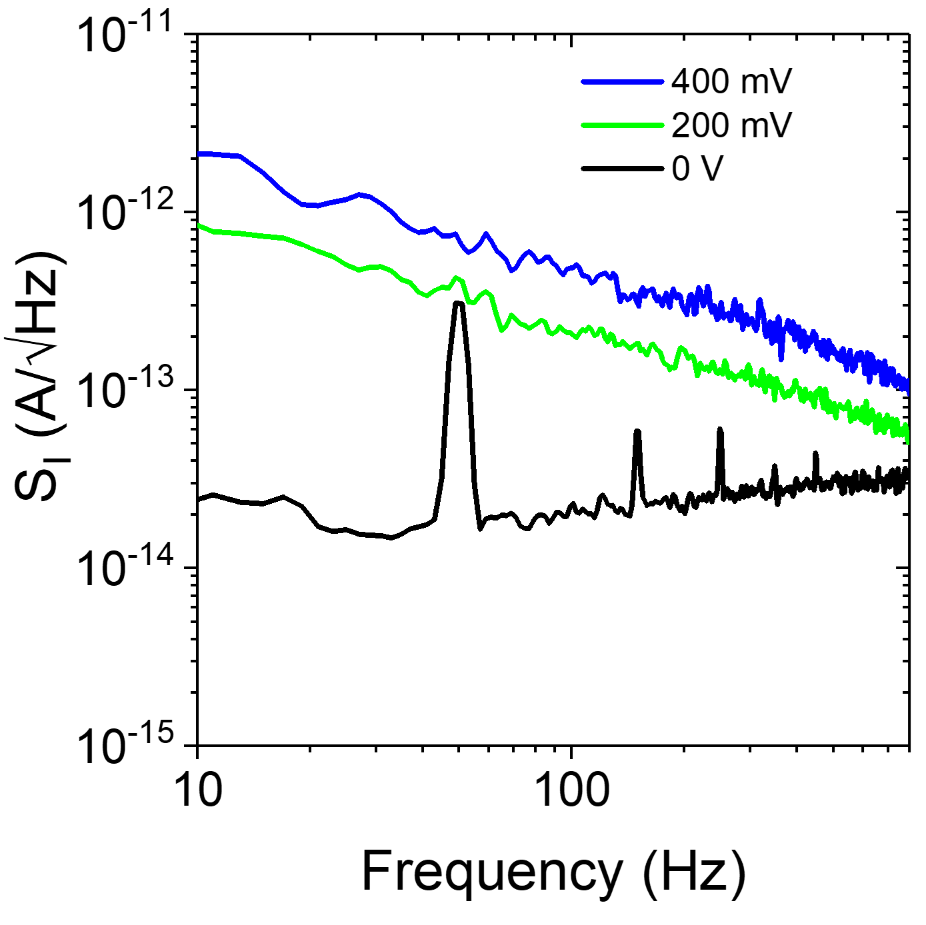


**Figure S 5 Noise Spectra**. Noise spectral current density acquired at 300 K under 3 different biases.

# Device for photoemission imaging

Photoemission imaging requires soft energy photons to minimize the spot size due to the chromaticity of the Fresnel zone plate used to focus the beam. Imaging relies on the tracking of the energy shift of a given core level, which therefore should present a binding energy below the photon energy (i.e., below 95 eV). In our case, three states are relevant: Hg 5d (BE=8.3 eV), Cd 4d (BE=11.4 eV), and Te 4d (BE=40.2 eV), see **Figure S 6**. The procedure to fabricate the planar *pn* junction is described in **Figure S 7**.


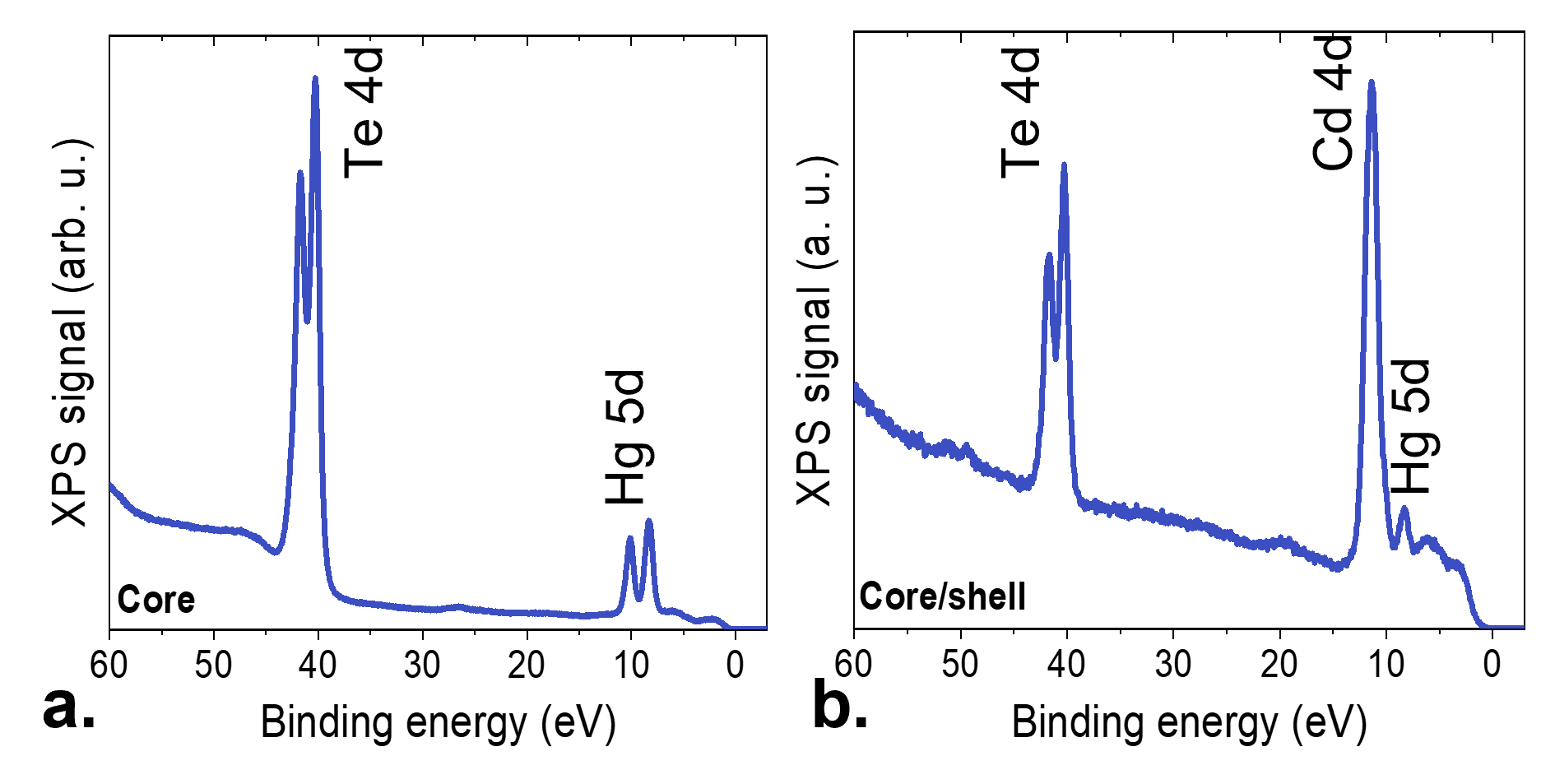


**Figure S 6 XPS spectra** for HgTe core and HgTe/CdS core, acquired at hv=95 eV.


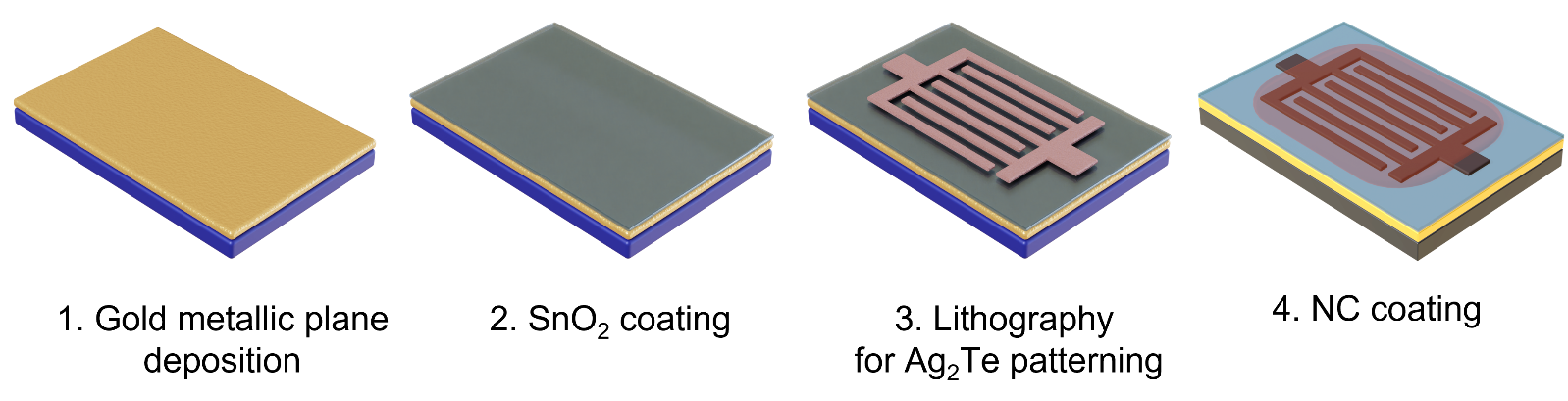


**Figure S 7 Fabrication of the planar pn junction** used for the photoemission imaging experiment.

# Integration of the diode on a dielectric mirror


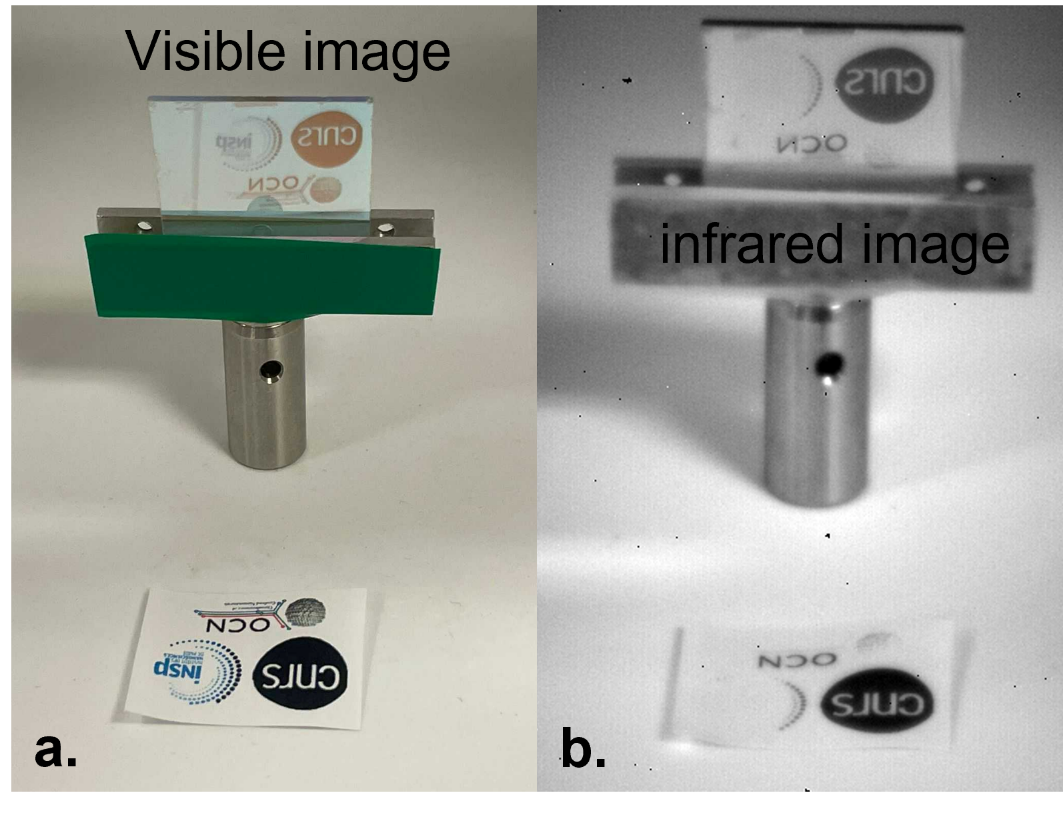


**Figure S 8 Dielectric cavity acting as infrared mirror**. Visible (CMOS-based) (a.) and infrared (InGaAs-based) (b.) image of the dielectric stack highlighting its behavior as a mirror.


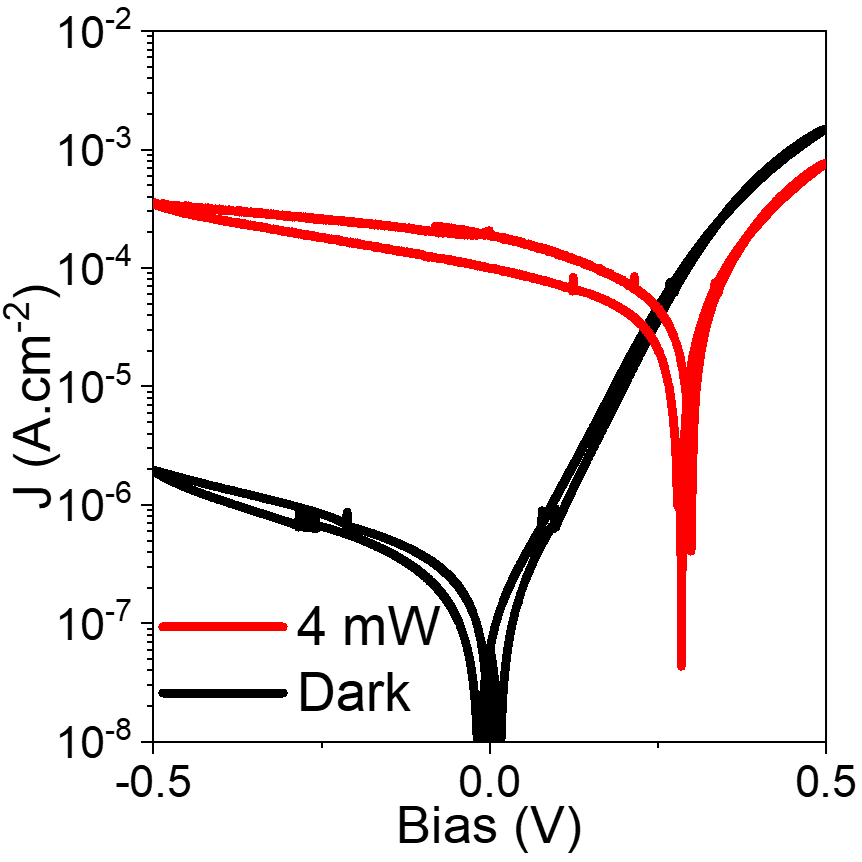


**Figure S 9 IV curve for the diode on dielectric mirror** in the dark and under illumination by a 4 mW laser diode at 1.55 µm.

**Figure S 10**a provides the model structure of the diode coupled to the Bragg mirror used for electromagnetic simulation. **Figure S 10**b-e provides the complex index of various layers. For HgTe/CdS and Ag_2_Te (due to the cation exchange) we assume that they behave as the HgTe core NCs. The refractive index/thickness of the Bragg are taken from *in-situ* measurements conducted during the growth.


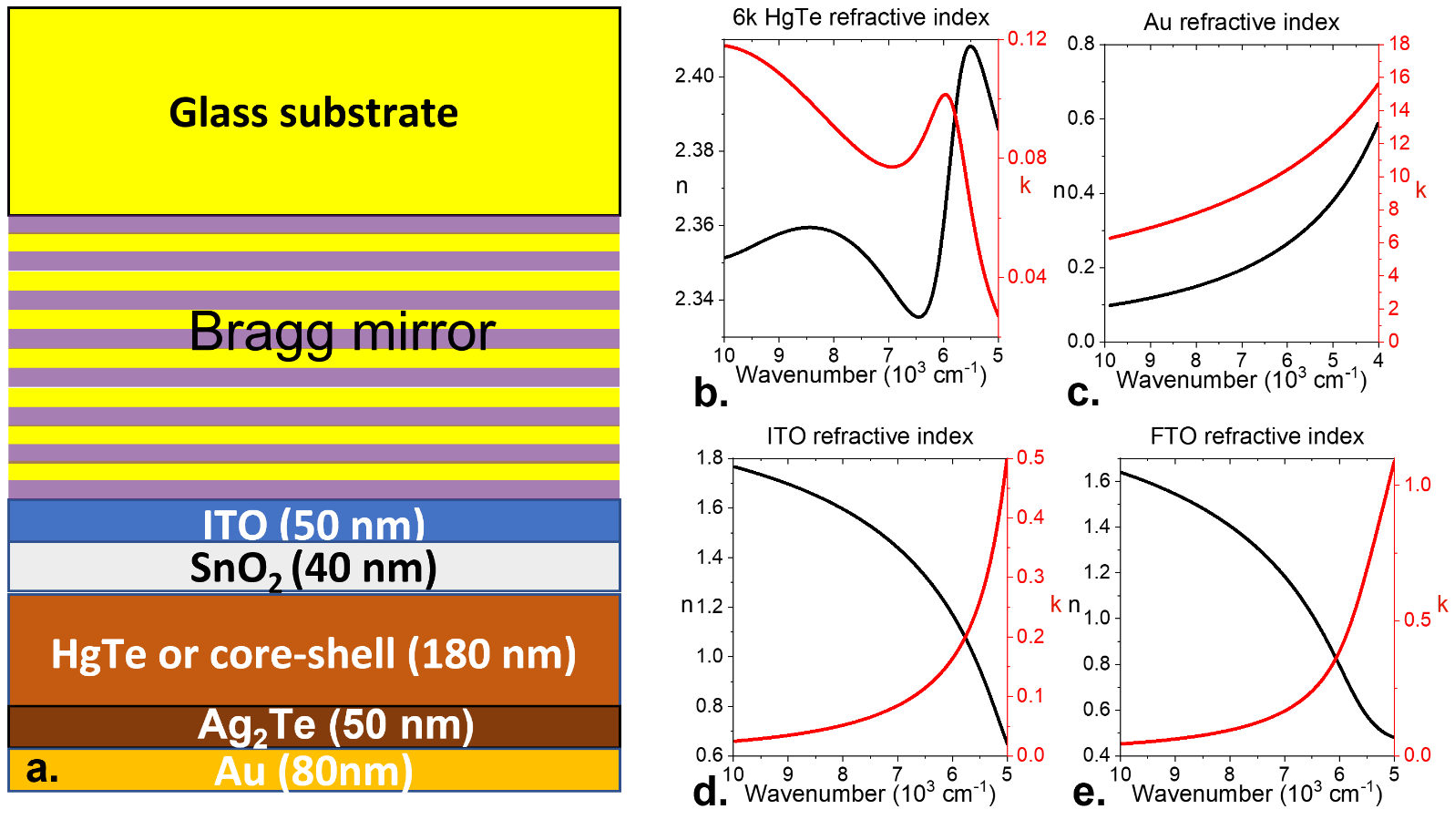


**Figure S 10 Electromagnetic modelling of the diode in cavity stack**. a. Schematic of the diode on dielectric mirror stack. Complex optical index (i.e., refractive index and extinction coefficient spectra) for HgTe NCs (b.), gold (c.), ITO (d.) and FTO (e.)


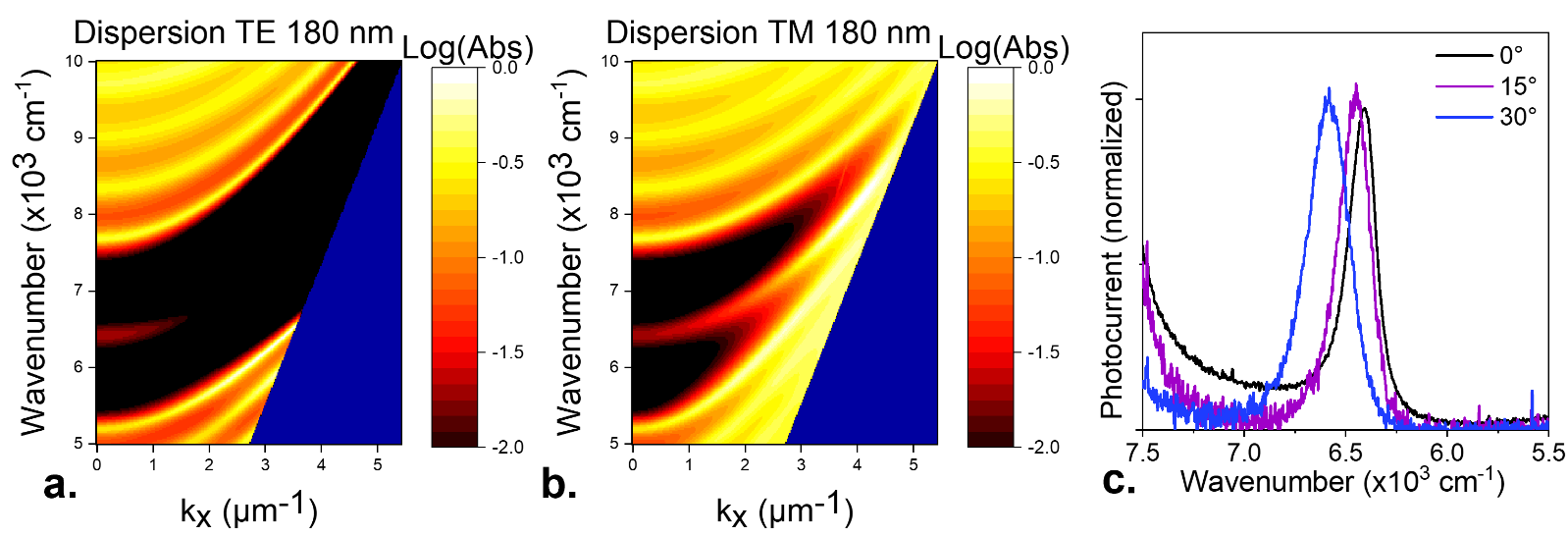


**Figure S 11 Dispersion map for the diode on Bragg stack**. (a). in TE polarization and (b.) in TM polarization. c. Experimental photocurrent for three angles

**Figure S 11** provides the dispersion map for the diode on Bragg along the two polarizations (**Figure S 11**a and b). Experimentally, it was observed that the cavity mode shifts toward higher energy when the sample is illuminated at an angle with respect to the normal incidence, which match the predicted dispersion.

JV curve of the diode on the dielectric mirror is given in **Figure S 9**. **Figure S 12** highlights the lack of correlation between the linewidth of the cavity mode and the pixel area.

Photocurrent (**Figure S 12**a) shows a lack of correlation between the pixel size and the mode linewidth. This claim is further supported by infrared reflectivity measurement (**Figure S 12**b), where the cavity mode spectra presents a quasi-constant linewidth as the spot size is tuned over 1 order of magnitude. The lack of dependence suggests that the two mirrors (the Bragg and the gold one) are parallel enough over the probed scale.^[6]^


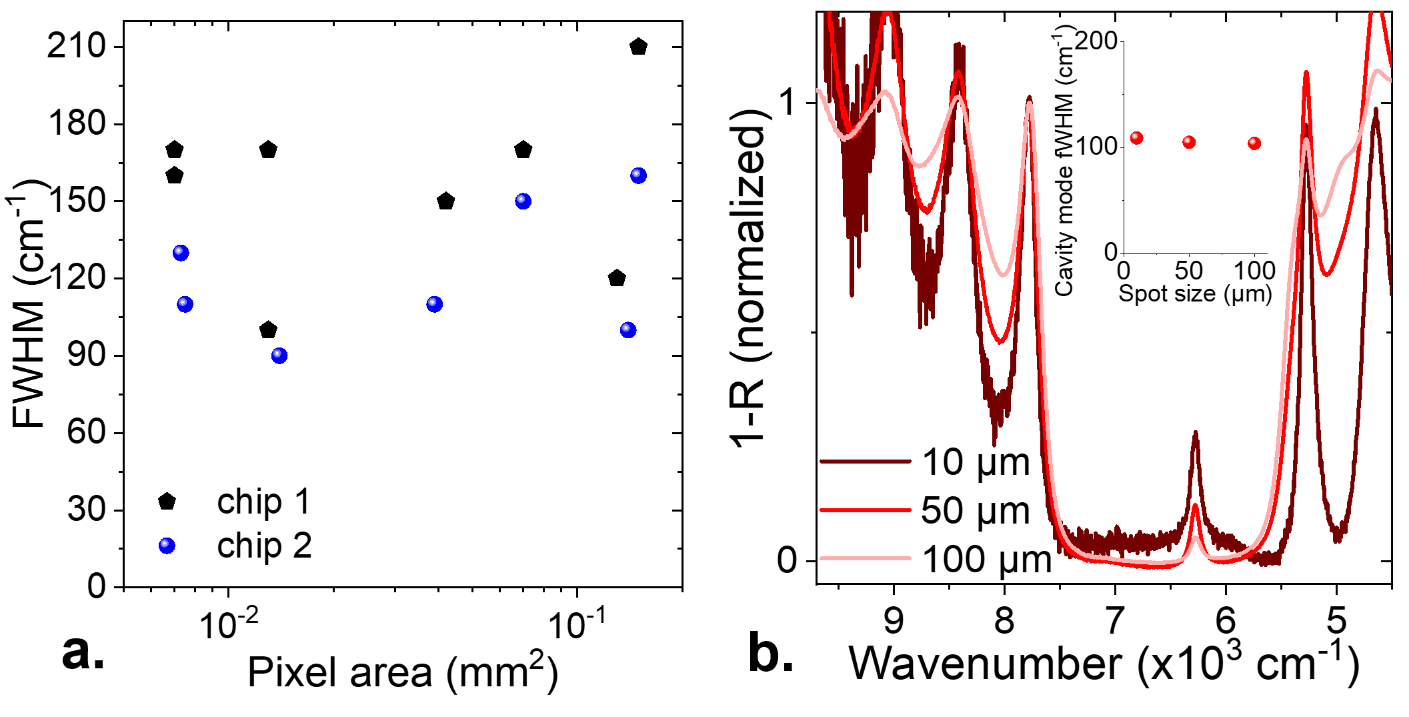


**Figure S 12 Linewidth of the cavity mode as a function of the pixel area**. a. Experimental FWHM of the cavity mode measured through the photocurrent spectra for various diode areas on two chips**.** b. Experimental absorbance spectra (measured through the estimation of 1-reflectivity) for three optical spot sizes. The inset provides the FWHM of the mode as a function of the optical spot size. The graphs stress the absence of correlation of the two quantities.

Conversely, the cavity mode appears strongly dependent on the diode absorption. The latter is tuned artificially using simulation, where the extinction coefficient is artificially reduced from its nominal value at the band edge (*k*=0.1),^[7]^ to an arbitrary low value of 10^-3^. The absorptions from the contact (gold and ITO) are kept unchanged for this simulation. As *k* gets reduced, the cavity mode gets narrower and gain intensity, see **Figure S 13**a and b.


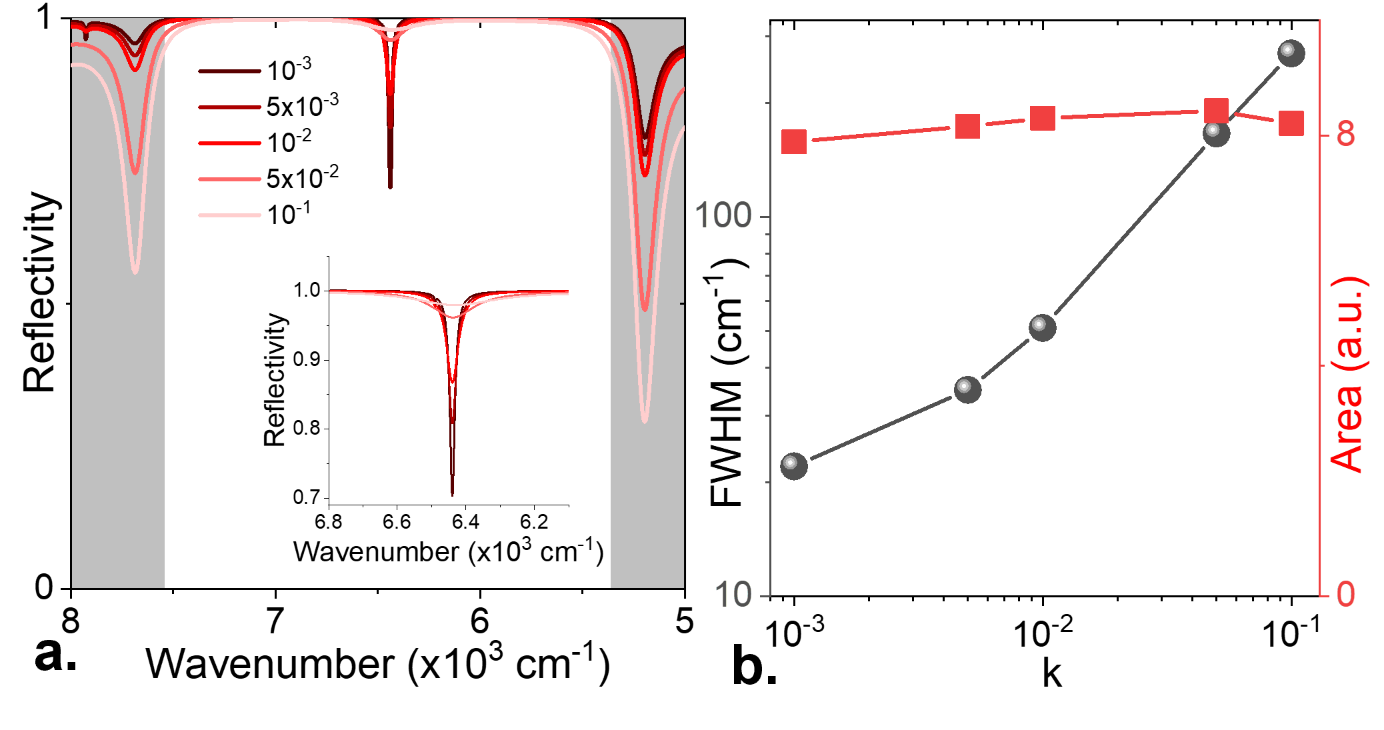


**Figure S 13 Effect of the cavity absorption on its linewidth.** a. Simulated reflectivity spectra for three various values of the extinction coefficient from the NCs (i.e., the HgTe/CdS together with the Hg cation exchanged Ag_2_Te layer). The inset is a zoom on the cavity mode. b. FWHM and area of the cavity mode as a function of the extinction coefficient from the NCs.

# REFERENCES

[1] S. Keuleyan, E. Lhuillier, P. Guyot-Sionnest, *J. Am. Chem. Soc.* **2011**, *133*, 16422.

[2] H. Zhang, R. Alchaar, Y. Prado, A. Khalili, C. Gréboval, M. Cavallo, E. Bossavit, C. Dabard, T. H. Dang, C. Abadie, C. Methivier, D. Darson, V. Parahyba, P. Potet, J. Ramade, M. G. Silly, J. K. Utterback, D. Pierucci, S. Ithurria, E. Lhuillier, *Chem. Mater.* **2022**, *34*, 10964.

[3] M. M. Ackerman, X. Tang, P. Guyot-Sionnest, *ACS Nano* **2018**, *12*, 7264.

[4] G. R. Savich, J. R. Pedrazzani, D. E. Sidor, S. Maimon, G. W. Wicks, *Appl. Phys. Lett.* **2011**, *99*, 121112.

[5] H. Liu, E. Lhuillier, P. Guyot-Sionnest, *J. Appl. Phys.* **2014**, *115*, 154309.

[6] E. Bossavit, D. Mastrippolito, C. Gureghian, A. Colle, D. De Pesseroey, M. Paye, K. Sergeeva, M. Cavallo, Y. Ma, A. Khalili, T. Gemo, Y. Prado, M. Hamieh, E. Dandeu, S. Ithurria, D. Pierucci, M. G. Silly, X. Lafosse, E. Lhuillier, *Nano Lett.* **2025**, *25*, 9485.

[7] P. Rastogi, A. Chu, T. H. Dang, Y. Prado, C. Gréboval, J. Qu, C. Dabard, A. Khalili, E. Dandeu, B. Fix, X. Z. Xu, S. Ithurria, G. Vincent, B. Gallas, E. Lhuillier, *Adv. Opt. Mater.* **2021**, *9*, 2002066.
